# Long noncoding RNA *Sox2ot* and transcription factor YY1 co-regulate the differentiation of cortical neural progenitors by repressing *Sox2*

**DOI:** 10.1038/s41419-018-0840-2

**Published:** 2018-07-23

**Authors:** Jennifer L. Knauss, Nan Miao, Seung-Nam Kim, Yanzhen Nie, Yuelin Shi, Tao Wu, Hugo Borges Pinto, Mary E. Donohoe, Tao Sun

**Affiliations:** 1 Department of Cell and Developmental Biology, Cornell University Weill Medical College, 1300 York Avenue, Box 60, New York, NY 10065 USA; 2 Center for Precision Medicine, School of Medicine and School of Biomedical Sciences, Huaqiao University, Xiamen, China 361021; 3 College of Korean Medicine, Dongguk University, Ilsandonggu, Goyangsi, 10326 Gyeonggido Korea; 4 School of Life Sciences and Technology, Shanghai Jiao Tong University, Shanghai, China 200240; 5 Burke Medical Research Institute, 785 Mamaroneck Avenue, White Plains, NY 10605 USA; 6 Department of Neuroscience, Department of Cell and Developmental Biology, Cornell University Weill Medical College, 1300 York Avenue, New York, NY 10065 USA

## Abstract

Long noncoding RNAs (lncRNAs) are emerging as key regulators of crucial cellular processes. However, the molecular mechanisms of many lncRNA functions remain uncharacterized. *Sox2ot* is an evolutionarily conserved lncRNA that transcriptionally overlaps the pluripotency gene *Sox2*, which maintains the stemness of embryonic stem cells and tissue-specific stem cells. Here, we show that *Sox2ot* is expressed in the developing mouse cerebral cortex, where it represses neural progenitor (NP) proliferation and promotes neuronal differentiation. *Sox2ot* negatively regulates self-renewal of neural stem cells, and is predominately expressed in the nucleus and inhibits Sox2 levels. *Sox2ot* forms a physical interaction with a multifunctional transcriptional regulator YY1, which binds several CpG islands in the *Sox2* locus in a *Sox2ot*-dependent manner. Similar to *Sox2ot*, YY1 represses NP expansion in vivo. These results demonstrate a regulatory role of *Sox2ot* in promoting cortical neurogenesis, possibly by repressing *Sox2* expression in NPs, through interacting with YY1.

## Introduction

Production of distinct types of neural progenitors and neurons is regulated by coding and noncoding RNAs in the mammalian cerebral cortex^1–5^. Long noncoding RNAs (lncRNAs), generally considered as transcripts of hundreds of nucleotides (nt) in length with little or no protein-coding potential, comprise a large proportion of the mammalian genome^6–8^. Though thousands of lncRNAs have been reported, relatively few have been mechanistically characterized. Those that have been characterized appear to work through a wide variety of mechanisms, including control of chromatin structure, transcription, mRNA processing, and translation^9,10^. Emerging studies have shown a specific and dynamic expression of lncRNAs in embryonic stem (ES) cells, tissue-specific stem cells, and progenitors in various species. However, the molecular mechanisms of lncRNA function in specific cells and tissues and their relationship to diseases remain unclear^11–13^.

*Sox2* is a pluripotency gene that maintains the stemness of human and rodent ES cells^14–16^. However, the molecular regulation of *Sox2* expression is incompletely understood. Several CpG islands are harbored in the *Sox2* locus, which cover and flank the *Sox2* gene. This suggests a possible role for CpG islands in *Sox2* regulation by attracting transcription factors for transcriptional initiation, or propagating transcriptional silencing via DNA methylation^17^. For instance, the *Sox2* promoter in ES cells is marked by permissive H3K4me3 histone marks, while the surrounding CpG islands are bivalently marked, indicating that the locus may also be poised for repression^18^. *Sox2* is also highly expressed in neural stem cells (NSCs) and neural progenitors (NPs) in the mouse embryonic cerebral cortex, maintains the populations of NSCs and NPs, and promotes proliferation^19,20^. *Sox2* expression is downregulated in postmitotic, differentiated neurons; however, the regulatory mechanisms governing this transition are incompletely understood^16,21–24^.

Interestingly, the lncRNA *Sox2* overlapping transcript (*Sox2ot*) is highly conserved among species and overlaps the *Sox2* gene in the genome^25–27^. *Sox2ot* appears to be transcribed from several transcription start sites and can be differentially spliced, thus generating several *Sox2ot* isoforms^25,28^. While the function of Sox2 is defined, the roles of *Sox2ot* in development, stem cell expansion, and differentiation are unknown.

In this study, we demonstrate that *Sox2ot* negatively regulates neural progenitor proliferation by interacting with the epigenetic regulator YY1 in the developing mouse cerebral cortex. Overexpression of *Sox2ot* causes a decrease in NPs with a concomitant increase in neurons, while *Sox2ot* knockdown results in increased NPs and a loss of differentiated neurons. There is a strong interaction between *Sox2ot* and YY1 in the nucleus of neuroectodermal cells. Furthermore, we found that YY1 binds to CpG islands at the *Sox2* locus and represses NP proliferation, and that this binding is dependent on *Sox2ot*. Thus, we propose that *Sox2ot* exerts its effects in NP development with its RNA-binding partner YY1 through negative regulation of *Sox2* in the developing cortex.

## Results

### *Sox2ot* and *Sox2* are coexpressed in the ventricular zone in the developing mouse cortex

**Fig. 1:**
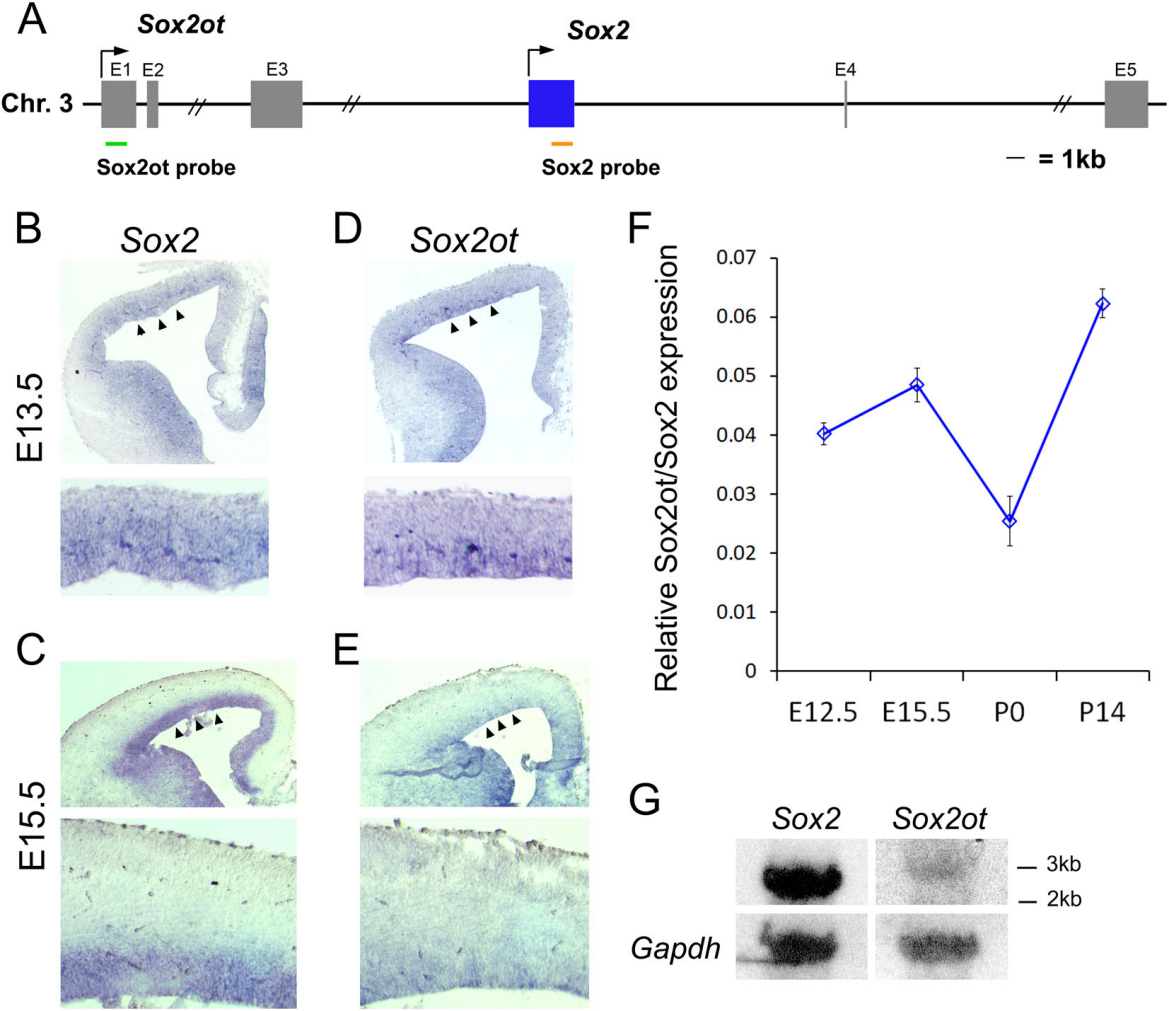
Characterization of *Sox2* and *Sox2ot* expression throughout cortical development. **a** Diagram of mouse *Sox2ot* locus. Green, *Sox2ot* probe; orange, *Sox2* probe. Chr, chromosome. Exon 1 (E1) to 5 (E5) are labeled. **b**–**e** Low-power (top panels) and high-power (bottom panels) images of in situ hybridization in the cerebral cortex of wild-type mice at E13.5 and E15.5. The ventricular zone expression of *Sox2* (arrowheads) and *Sox2ot* (arrows) is highlighted. **f** Ratio of *Sox2ot*/*Sox2* expression in cDNA from the dorsal cortex at various developmental stages detected by droplet digital quantitative PCR (ddPCR). *n* = 3 repeats. **g** Northern blot detecting *Sox2* and *Sox2ot* in the RNA extracted from the E13.5 dorsal cortex (upper panels). *Gapdh* is shown as a loading control (lower panels)

The main isoform of *Sox2ot* (GenBank Accession No. BC057611) contains five exons spanning >100 kilobases (kb) of the mouse genome, though the final mRNA is spliced to about 3 kb. *Sox2*, a single-exon transcript of about 2.5 kb, is located within one of the large introns of *Sox2ot* (Fig. 1a). None of the known isoforms of *Sox2ot* contain any overlapping exonic sequence with *Sox2*^25^. Given the overlapping genomic orientation of *Sox2* and *Sox2ot*, we performed in situ hybridization on embryonic mouse cerebral cortices to examine the expression patterns of each of these genes. Both *Sox2* and *Sox2ot* were expressed in the ventricular zone (VZ) and subventricular zone (SVZ) of cortices at embryonic day 13.5 (E13.5) and E15.5, which correspond to the active stages of neural progenitor expansion (Fig. 1b–e). The expression level of *Sox2ot* was not as robust as *Sox2*. Expression of *Sox2* and *Sox2ot* was reduced in postnatal day 0 (P0) cortices, corresponding with a reduction in the NP population (Supplementary Figure S1A). Moreover, sense probes for *Sox2* and *Sox2ot* lacked specific staining in E13.5 cortices (Supplementary Figure S1B).

To establish a more quantitative picture of *Sox2* and *Sox2ot* expression levels, we performed droplet digital quantitative PCR (ddPCR) to test their mRNA copy numbers in the dorsal cortex. From E12.5 to P14, the mRNA copy numbers of *Sox2* and *Sox2ot* showed a reduced trend (Supplementary Figure S1C and D). Moreover, *Sox2* was expressed at a higher level than *Sox2ot* at all developmental stages tested, and the ratio of *Sox2ot*/*Sox2* expression was slightly increased, suggesting an elevated expression of *Sox2ot* through development (Fig. 1f).

To further verify the expression levels and sizes of *Sox2* and *Sox2ot* transcripts, we performed Northern blot analyses on RNA extracted from E13.5 mouse dorsal cortices (Fig. 1g). As expected, *Sox2* was expressed at a higher level than *Sox2ot*, which reflected the expression observed in the analyses of ddPCR (Fig. 1b–f). Both *Sox2* and *Sox2ot* transcripts were detected at the expected sizes, 2.5 kb and 3 kb, respectively (Fig. 1g). Taken together, these experiments establish that *Sox2ot* is expressed in the VZ, where NPs reside in the developing cortex.

### *Sox2ot* overexpression causes a reduction of neural progenitors

**Fig. 2:**
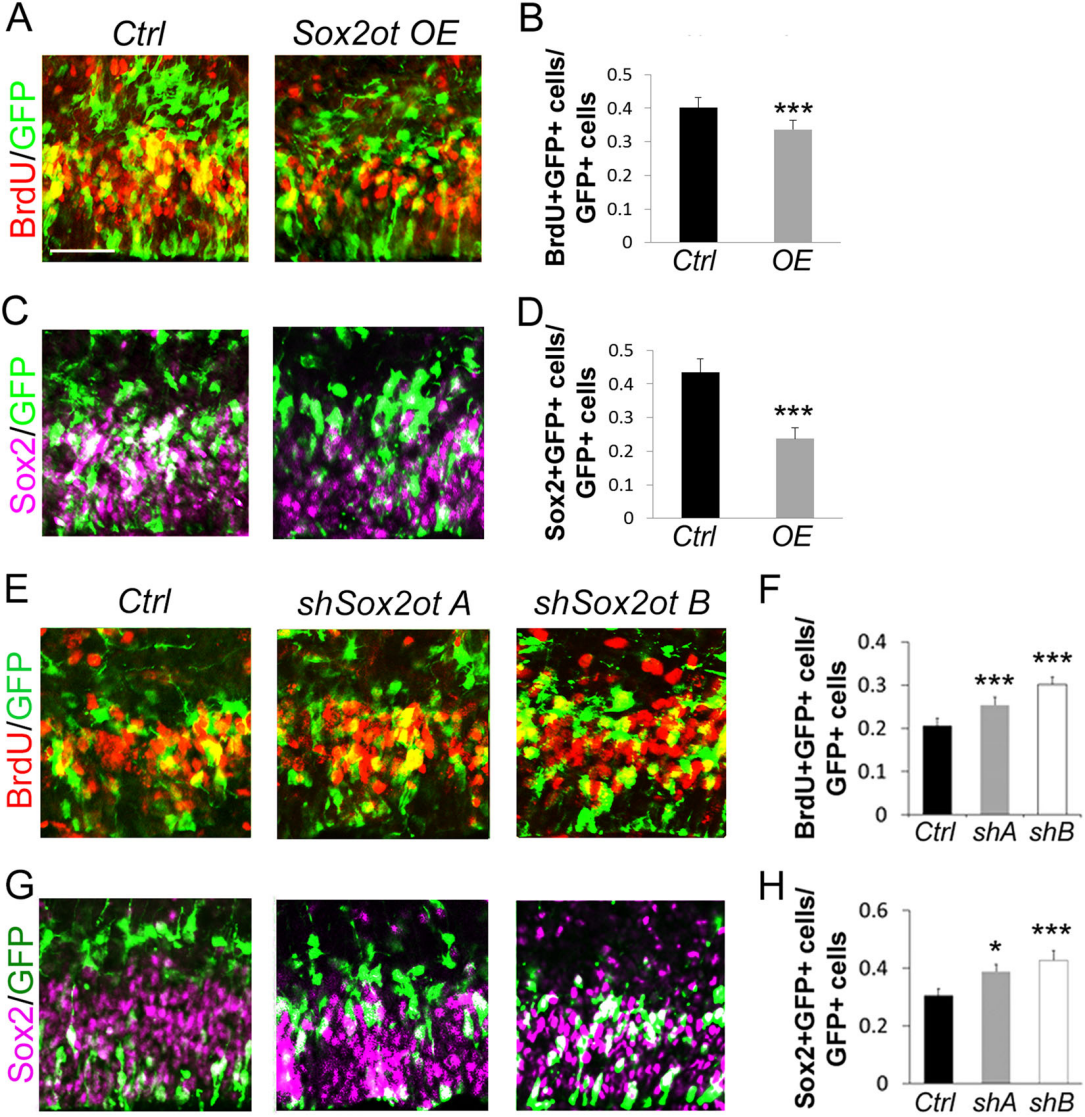
Manipulation of *Sox2ot* affects the development of neural progenitors. **a**–**d** Electroporation of ectopic *Sox2ot (OE)* at E13.5 for analysis at E14.5 significantly decreased the number of BrdU-incorporating or Sox2^+^ cells co-labeled with GFP in the cortex, compared to the control (*Ctrl*). **e**–**h** Electroporation of shRNAs against *Sox2ot* (*shSox2ot A* and *shSox2ot B*) at E13.5 for analysis at E14.5 significantly increased the number of BrdU-incorporating or Sox2^+^ cells co-labeled with GFP in the cortex. Yellow and white cells indicate co-labeled cells. Data are presented as mean±SD; *n* ≥ 5 sections from at least four different brains; *p* values in relation to the empty vector control (*p *<* 0.05, ***p *<* 0.001). Scale bar=50 µm

To explore the function of *Sox2ot* in NPs, we first overexpressed *Sox2ot* by in utero electroporation (IUE) at E13.5 and collected the brains at E14.5 for analysis. A bromodeoxyurindine (BrdU) pulse was given 1 h before tissue collection to label the dividing cells in the S-phase in a cell cycle. Electroporation in the mouse embryonic cortex will first label neural progenitors in the VZ. To avoid biased analyses, all quantification in this study was based on calculating the ratio of marker^+^/GFP^+^ versus GFP^+^. Measures of the general number of NPs with BrdU incorporation (BrdU^+^/GFP^+^ cells versus GFP^+^ cells) and Sox2 staining (Sox2^+^/GFP^+^ cells versus Sox2^+^ cells) were both decreased in *Sox2ot* overexpression (*OE*) cortices compared to controls (*Ctrl*) (Fig. 2a–d). The reduction in the numbers of Sox2^+^ cells in GFP^+^ cells suggests the potential for direct negative regulation of *Sox2* by *Sox2ot*. Two specific NP populations, radial glia cells (RGCs) and intermediate progenitors (IPs) that can be labeled by Pax6 and Tbr2, respectively, also showed a decrease in the *Sox2ot OE* cortices compared to controls, by calculating the ratios of Pax6^+^/GFP^+^ and Tbr2^+^/GFP^+^ cells versus GFP^+^ cells (Supplementary Figure S2A–D).

To confirm these results quantitatively, we dissected the GFP-positive area of the cortex 24 h after E13.5 electroporation and extracted RNA for real-time quantitative reverse transcription PCR (qRT-PCR) (Supplementary Figure S3). As expected, *Sox2ot* was upregulated in *Sox2ot OE* brains, while *Sox2* was downregulated, reflecting the immunostaining results (Supplementary Figure S3A). *Pax6* and *Tbr2* RNA levels also mirror protein-level changes upon *Sox2ot OE* (Supplementary Figure S3B).

Moreover, we hypothesized that the loss of NPs upon *Sox2ot OE* could be explained by cell death or by differentiation. We then examined cell death using activated Caspase3 as a marker. In E14.5 brains, activated Caspase3 expression was not significantly altered due to *Sox2ot* overexpression, indicating that cell death does not explain the loss of NPs (Supplementary Figure S2E and F). These results demonstrate that *Sox2ot OE* represses NP expansion in both RGCs and IPs, and that this function is not carried out through cell death.

### *Sox2ot* knockdown increases neural progenitors

To further examine the effect of *Sox2ot* on NP expansion, we applied a knockdown approach using short hairpin RNAs (shRNAs) for *Sox2ot*, named as *shSox2otA (shA)* and *shSox2otB (shB)* (Supplementary Figure S4A). In cell culture testing, both *shA* and *shB* significantly reduced *Sox2ot* expression, though *shB* had a slightly stronger effect (Supplementary Figure S4B). We again used the IUE technique to knock down *Sox2ot* in the developing cortex. Both BrdU incorporation and Sox2 staining increased in *shSox2ot* brains compared to control brains (Fig. 2e–h). The specific progenitor population markers for RGCs and IPs also showed increases upon *Sox2ot* knockdown (Supplementary Figure S5). In each case, the increase in NPs is slightly larger for *shB* than *shA*, though both are significant, reflecting the strength of knockdown observed in cell culture experiments (Fig. 2; Supplementary Figure S4B). Our qRT-PCR of electroporated brains also confirmed these results, showing *Sox2ot* knockdown, *Sox2* increase, and upregulation of both *Pax6* and *Tbr2* upon electroporation of *shSox2otA* (Supplementary Figure S4C and D). These observations further support the functionality of *Sox2ot* as a negative regulator in NP expansion in the developing cortex.

### *Sox2ot* affects neural differentiation

**Fig. 3:**
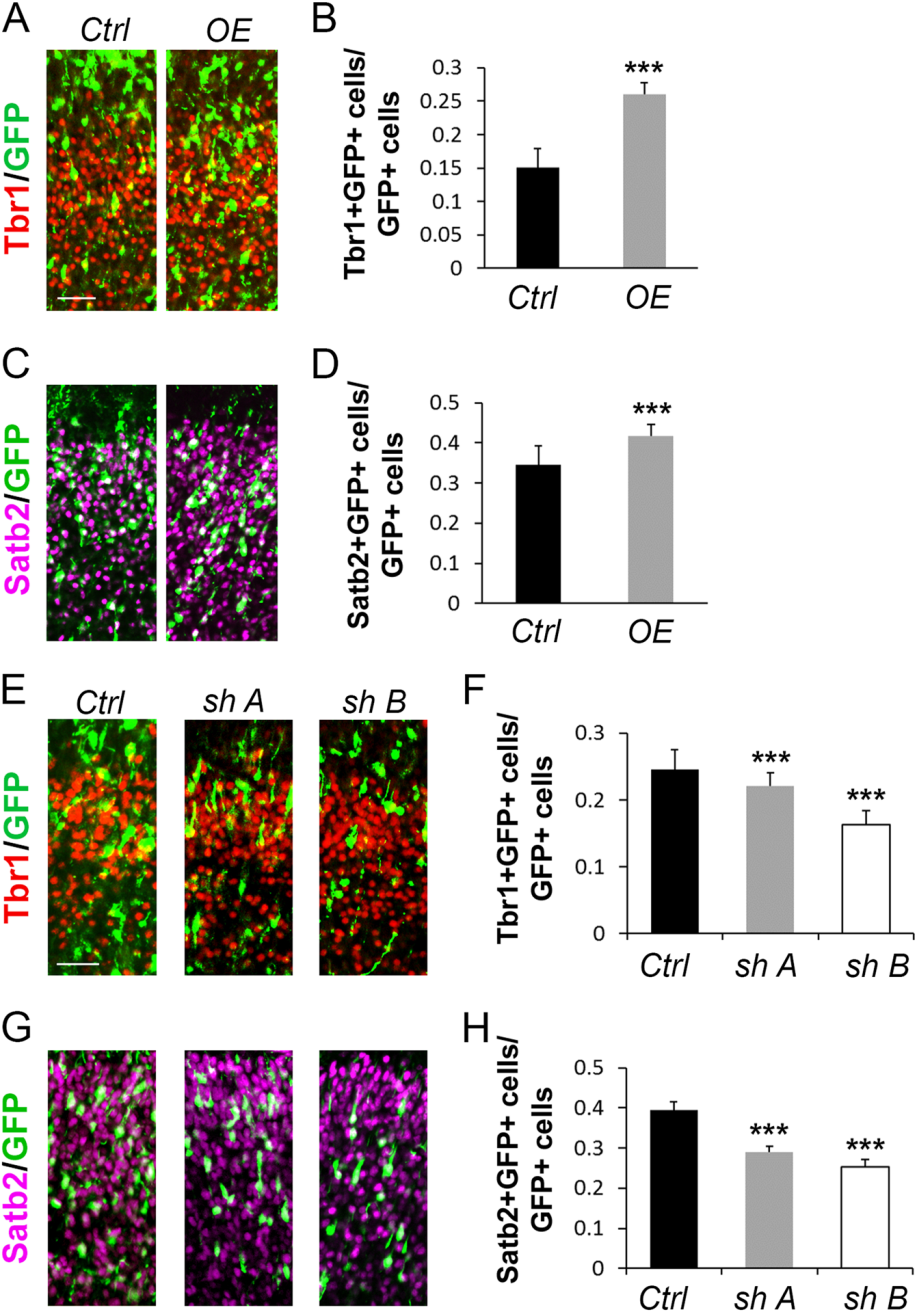
Loss of neural progenitors caused by altered *Sox2ot* is due to early differentiation. **a**–**d** Electroporation of ectopic *Sox2ot (OE)* at E13.5 for analysis at E17.5 significantly increased the number of Tbr1^+^ or Satb2^+^ cells co-labeled with GFP in the cortex, compared to the control (*Ctrl*). **e**–**h** Electroporation of shRNAs against *Sox2ot* (*shSox2ot A* and *shSox2ot B*) at E13.5 for analysis at E17.5 significantly decreased the number of Tbr1^+^ or Satb2^+^ cells co-labeled with GFP in the cortex. Yellow and white cells indicate co-labeled cells. Data are presented as mean ± SD; *n* ≥ 5 sections from at least four different brains; *p* values in relation to the empty vector control (***p *<* 0.001). Scale bars: 50 µm

To determine the effects of *Sox2ot OE* on the differentiation of NPs, we electroporated the *Sox2ot OE* construct at E13.5 and collected brain tissues 4 days after electroporation at E17.5, thus allowing more time for *Sox2ot* to exert its effects on neurogenesis. Two markers for differentiated neurons, Tbr1 and Satb2, which label early-born and late-born neurons, respectively, each showed an increase with *Sox2ot OE* (Fig. 3a–d). This expansion of differentiated neurons accounts for the loss of NPs observed in 1-day electroporations, suggesting an early differentiation (Fig. 2a–d). These results were confirmed at the RNA level for *Tbr1* by qRT-PCR of RNA extracted from electroporated cells in the cortex (Supplementary Figure S3B).

We next examined the effect of *Sox2ot* knockdown on neuron populations and found that both Tbr1 and Satb2 expression was decreased, which is opposite to the effect of *Sox2ot OE* (Fig. 3e–h). Decreased neurogenesis corresponds to the increase in the neural progenitor pool upon *Sox2ot* knockdown (Fig. 2e–h). The observed decrease in Tbr1 was confirmed by qRT-PCR on RNA purified from electroporated brain cells (Supplementary Figure S4D). Collectively, these results establish a role for *Sox2ot* in the NP choice between proliferation and differentiation.

### *Sox2ot* is localized to the nucleus in neuroectodermal cells

**Fig. 4:**
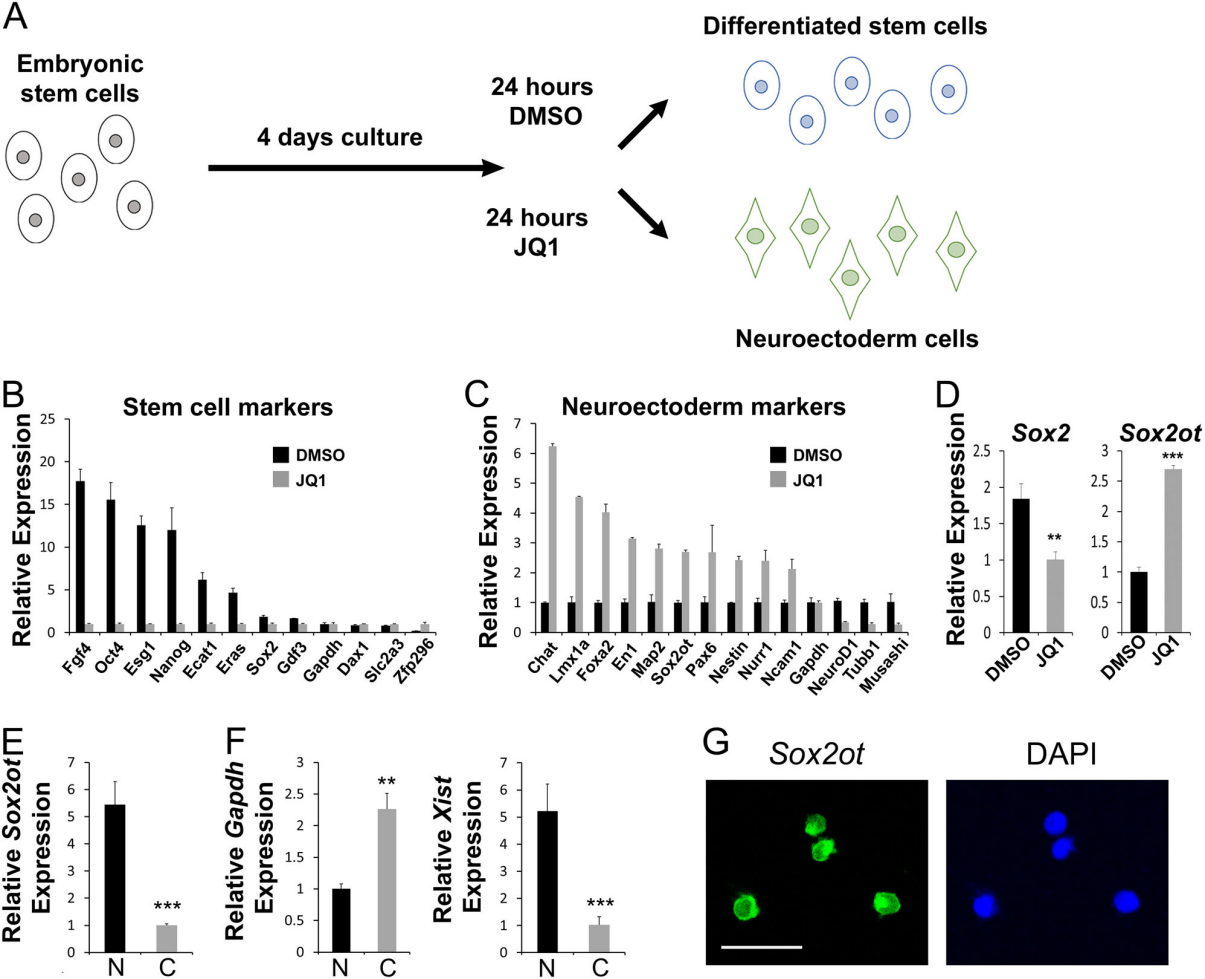
*Sox2ot* is localized to the nucleus in neuroectodermal cells and neural progenitors. **a** Diagram of the mouse embryonic stem (ES) cell culture protocol for neuroectoderm differentiation. **b**, **c** Real-time RT-PCR detecting the relative expression of markers for stem cells (**b**) and neuroectoderms (**c**) in JQ1 (gray) and DMSO- (black) treated ES cells. **d** Real-time RT-PCR detecting *Sox2* and *Sox2ot* in JQ1 and DMSO-treated ES cells. **e**, **f** Real-time RT-PCR detecting *Sox2ot*, *Gapdh*, and *Xist* expression in nuclear (N, black) and cytoplasmic (C, gray) fractions of neuroectodermal cells differentiated from female mouse LF2 ES cells. **g** Nuclear expression of *Sox2ot* in cultured mouse neural progenitors derived from E12.5 cortex detected by RNA fluorescent in situ hybridization (FISH). Data are presented as mean±SD; *n* = 3 for all real-time RT-PCR; *p* values in relation to the control (**p < 0.01, ***p *<* 0.001). Scale bars=20 µm

Having established that *Sox2ot* plays a role in regulating cortical neurogenesis, we sought to characterize how *Sox2ot* exerts its function. Because there are a wide variety of cell types present in the developing cortex, we turned to cultured and relatively homogeneous mouse ES cells (mESCs) and their derived neuroectodermal cells. mESCs were cultured for 4 days and then treated for 24 h with either DMSO vehicle or the small molecule JQ1, a BET protein inhibitor shown to cause differentiation to a neuroectodermal fate^29^ (Fig. 4a). To confirm the identity of mESCs after treatment, we performed qRT-PCR for various stem cell and neuronal markers. mESCs treated only with DMSO showed enrichment for stem cell markers, including *Fgf4*, *Oct4*, and *Nanog*, while mESCs treated with JQ1 expressed higher levels of neuroectodermal markers, such as *Nestin*, *Pax6*, and *Foxa2* (Fig. 4b, c). Notably, markers of more mature neurons, *NeuroD1* and *Tubb1*, were not enriched in the neuroectodermal cells, further validating their NP identity (Fig. 4c). Moreover, *Sox2* and *Sox2ot* were differentially expressed between differentiated stem cells and neuroectodermal cells, with *Sox2* decreased in neuroectodermal cells while *Sox2ot* increased, reflecting the changes in *Sox2ot* and *Sox2* expression observed in vivo (Fig. 1f), and indicating that *Sox2ot* may be specific to the differentiation of the neural lineage (Fig. 4c, d).

As lncRNAs can function in both the nucleus and the cytoplasm, we next determined the subcellular localization of *Sox2ot*. Lysis of cells followed by low-speed centrifugation pellets the relatively large nuclei intact, while leaving the cytoplasm as the supernatant, allowing reliable separation of nuclear and cytoplasmic RNAs. Using RNA extracted from neuroectodermal cells differentiated from female ES cells, we performed qRT-PCR for *Sox2ot* and determined that it is significantly enriched in the nuclear fraction (Fig. 4e). We also measured *Gapdh* and *Xist* as cytoplasmic and nuclear controls, respectively, and found that each of them was highly expressed in the expected fraction (Fig. 4f). Moreover, we examined *Sox2ot* expression in cultured mouse neural progenitors derived from E12.5 cortex using RNA fluorescent in situ hybridization (FISH). *Sox2ot* was mostly expressed in the nucleus (Fig. 4g).

These results demonstrate *Sox2ot* nuclear localization, suggesting that *Sox2ot* may play roles in transcriptional and epigenetic regulation of the switch in NP fate choice.

### *Sox2ot* binds YY1 in neuroectodermal cells

**Fig. 5:**
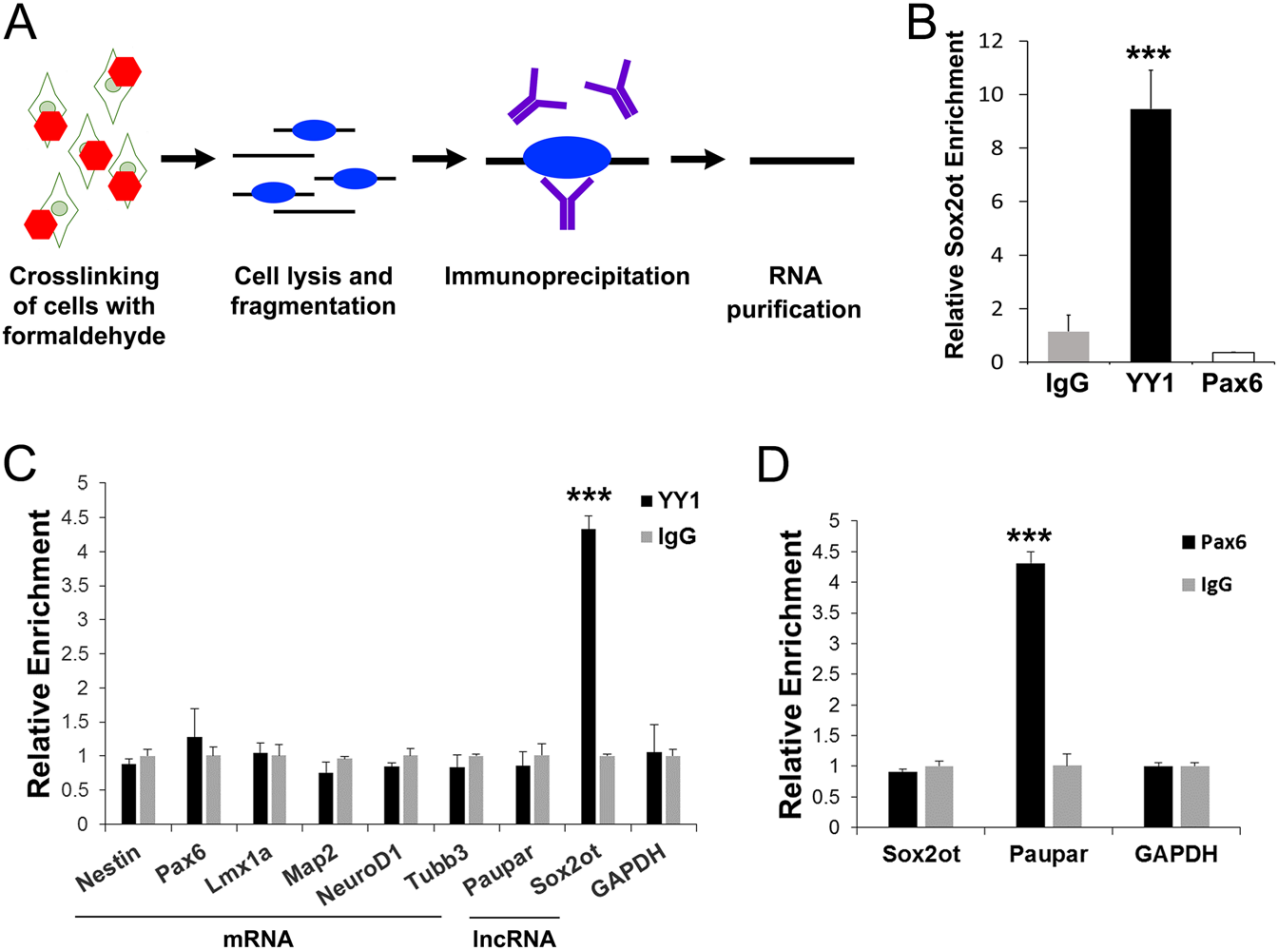
*Sox2ot* binds YY1 in an RNA immunoprecipitation (RIP) assay. **a** Schematic summary of the RIP protocol. **b** RIP with YY1, Pax6, or control antibody. Relative *Sox2ot* enrichment detected by real-time RT-PCR. **c** Real-time RT-PCR detection of various brain mRNAs and lncRNAs in YY1 RIP RNA. **d** Real-time RT-PCR detection of positive control *Paupar* lncRNA in Pax6 RIP RNA. Data are presented as mean± SD; *n* = 3 individual experiments for all real-time RT-PCR; *p* values in relation to the control (***p *<* 0.001)

In some cases, the simple act of transcription constitutes lncRNA functionality, while in other cases, the lncRNA interacts with protein partners to exert its function^30,31^. After finding *Sox2ot* in the nucleus, we asked whether *Sox2ot* might interact with any proteins to repress *Sox2* expression. Using a candidate approach with several proteins previously shown to bind lncRNAs and to regulate target gene expression, we performed RNA immunoprecipitation (RIP) on neuroectodermal cells (Fig. 5a). The candidates included YY1, which is important for tethering *Xist* lncRNA to the X chromosome during X inactivation^32^; CTCF interacts with lncRNAs during imprinting and is a protein-interacting partner of YY1^33,34^; and Pax6 interacts with lncRNA *Paupar* in neuroblastomal cells^35^. We found that *Sox2ot* was highly enriched by YY1 pulldown, while CTCF and Pax6 showed no significant enrichment over IgG control (Fig. 5b, data not shown). To ensure that the RIP experiment was specific, we tested the pulldown of several neural mRNAs such as NeuroD1 and other lncRNAs (e.g., Paupar) by YY1 and found significant enrichment only of *Sox2ot* (Fig. 5c). As a positive control, we also tested the pulldown of the known Pax6-bound lncRNA *Paupar*, and found its significant enrichment with Pax6 (Fig. 5d)^35^. These results demonstrate a *Sox2ot* binding partner and give insight into the possible mechanisms of *Sox2ot* action through the multiple known functions of YY1.

### YY1 binds CpG islands in the *Sox2* locus

**Fig. 6:**
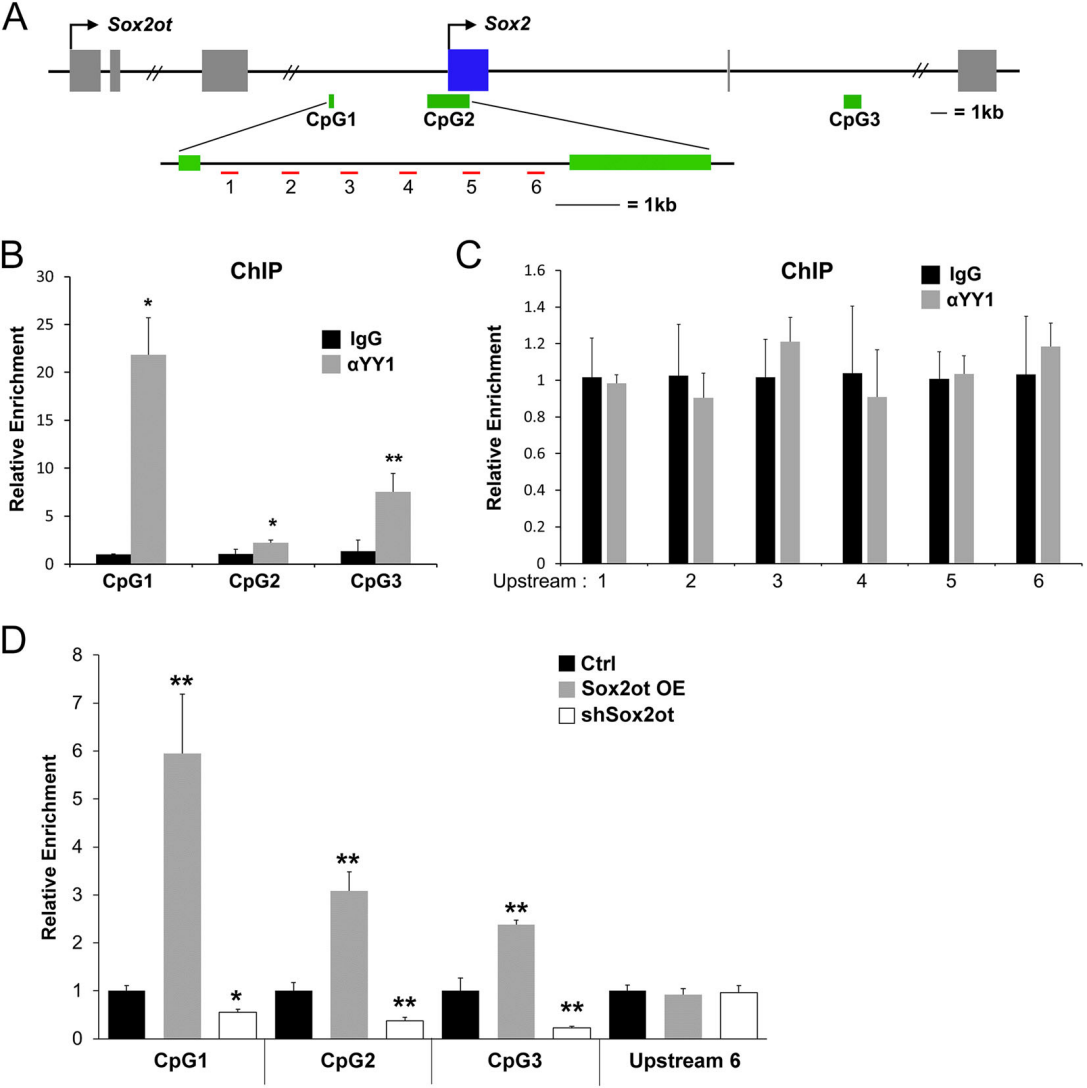
YY1 binds *Sox2* CpG islands in neuroectodermal cells. **a** Diagram of the *Sox2ot* locus demonstrating the location of CpG islands around the *Sox2* locus and primer locations in the upstream region of *Sox2*. **b**, **c** Chromatin immunoprecipitation (ChIP) with α-YY1 or control antibody. Relative enrichment of *Sox2* locus locations detected by real-time RT-PCT. **d** ChIP with α-YY1 or control antibody from the dissected electroporated region of mouse cortices that were electroporated with *Sox2ot OE*, *shSox2ot*, or control (*Ctrl*) vectors. Relative enrichment of *Sox2* locus locations detected by real-time RT-PCT. Data are presented as mean±SD; *n* = 3 different brain samples for all real-time RT-PCR; *p* values in relation to the control (*p < 0.05, **p < 0.01)

A subset of lncRNAs function by the *cis*-regulation of target genes near the same genomic locus^36^. Due to the overlapping genomic organization of the *Sox2*/*Sox2ot* locus (Fig. 1a), the similar expression patterns of *Sox2* and *Sox2ot* (Fig. 1b–e), the negative effects of *Sox2ot* on Sox2 expression (Fig. 2c, d, Supplementary Figure S3A), and the presence of favorable CpG island binding sites in the *Sox2* locus (Fig. 6a), we hypothesized that *Sox2ot* and its binding partner YY1 might function through repression of *Sox2*. Thus, we performed chromatin immunoprecipitation (ChIP) on chromatin in neuroectodermal cells to determine if YY1 could be found at the *Sox2* locus (Fig. 6a). Indeed, YY1 was found to bind at each of the three CpG islands in the *Sox2* locus (Fig. 6b). YY1 was not detected in the non-CpG island upstream region of the *Sox2* transcription start site (Fig. 6c).

To address the dependence of YY1 binding on *Sox2ot*, we performed ChIP of YY1 binding at each *Sox2* CpG island under *Sox2ot* overexpression or knockdown conditions. Upon overexpression of *Sox2ot*, YY1 binding at each CpG island was enriched, while knockdown of *Sox2ot* depleted YY1 binding (Fig. 6d and Supplementary Figure S6). As a control, we also tested YY1 binding at upstream region #6, and found no effect from *Sox2ot* manipulation (Fig. 6d). These results support a direct regulatory role for *Sox2ot* and YY1 in repression of *Sox2* in NPs.

### *YY1* knockdown causes an increase in neural progenitors

**Fig. 7:**
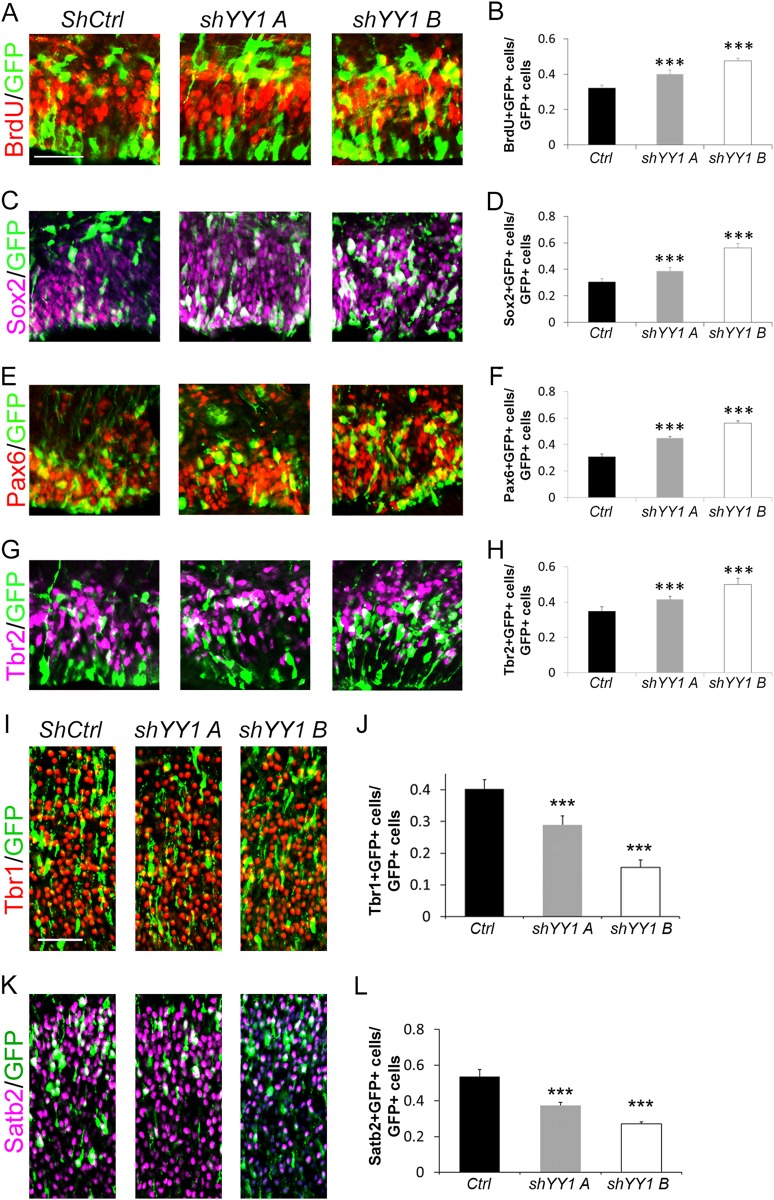
*YY1* knockdown increases neural progenitors. **a**–**h** Electroporation of shRNAs against *YY1* (*shYY1 A* and *shYY1 B*) at E13.5 for analysis at E14.5 significantly increased the number of BrdU-incorporating or Sox2^+^, Pax6^+^, or Tbr2^+^ cells co-labeled with GFP in the cortex, compared to the scrambled shRNA control (*ShCtrl*). **i**–**l** Electroporation of shRNAs against *YY1* at E13.5 for analysis at E17.5 significantly decreased the number of Tbr1^+^ or Satb2^+^ cells co-labeled with GFP in the cortex. Yellow and white cells indicate co-labeled cells. Data are presented as mean±SD; *n* ≥ 5 sections from at least four different brains for all electroporations; *p* values in relation to the scrambled shRNA control (***p < 0.001). Scale bar=50 µm

To further validate the functional relationship between *Sox2ot* and YY1, we performed IUE to knock down *YY1* in developing cortices using two different shRNAs, *shYY1A* and *shYY1B* (Fig. 7; Supplementary Figure S3C). We examined NPs at E14.5, 1 day after IUE, and found that the general progenitor cell markers BrdU and Sox2, both increased upon *YY1* knockdown (Fig. 7a–d). The specific RGC and IP markers Pax6 and Tbr2 also showed increased expression in *shYY1* cortices (Fig. 7e–h). We next examined the changes in cortical neurogenesis due to *YY1* knockdown using differentiated neuronal markers Tbr1 and Satb2. We found that both early- and late-born neurons were decreased (Fig. 7i–l).

Furthermore, changes in both the neural progenitor and mature neuronal populations caused by *YY1* knockdown were confirmed by RT-PCR of RNA from electroporated brains (Supplementary Figure S4C and D). These results are very similar to those observed upon *Sox2ot* knockdown in vivo (Figs. 2 and 3, Supplementary Figure S4C and D). We also performed RT-PCR for *YY1* in RNA extracted from brains electroporated with *Sox2ot OE* and *shSox2ot* to ensure that the observed phenotypes are not due to changes in *YY1* expression upon *Sox2ot* manipulation (Supplementary Figure S3C). Altogether, these results further support the functional relationship between *Sox2ot* and YY1.

## Discussion

As lncRNAs are continually annotated and validated, it has become clear that they are potent regulators of many cell processes. However, the mechanistic explorations of lncRNA functions are lacking. Here, we characterized the function of lncRNA *Sox2ot* in the developing mouse cerebral cortex through in vivo methods, determining that it promotes the differentiation of NPs into neurons, and suppresses the expansion of NPs. Using a mouse ES cell culture system, we also investigated the mechanism of *Sox2ot* regulation in neuroectodermal cells and found that it is active in the nucleus and interacts with the multifunctional transcription factor YY1. We demonstrated YY1 binding to CpG islands in the *Sox2* locus and provided evidence that this binding is mediated by *Sox2ot* and provides direct *cis*-regulation of *Sox2* expression in NPs (Supplementary Figure S7). Our results characterize *Sox2ot* function and give insight into the mechanism of a specific lncRNA, while providing support for the general archetype of lncRNA function as a scaffold for protein recruitment.

In the developing cerebral cortex, the balance between progenitor pool maintenance and neurogenesis is a highly complex and tightly regulated process^37^. RGCs are the main neural progenitor pool, have the ability to self-renew, generate a secondary IP pool, or directly generate neurons. IPs also proliferate transiently to expand the progenitor pool before generating neurons. As neurons are generated, they migrate outward into the cortex to form layers in an inside-out manner, such that the earliest-born neurons lie close to the inner surface of the cortex, while later-born neurons migrate past to populate the outer layers^38^. Errors in this intricate process have severe implications for proper cortex formation and function^39–41^.

In this study, we demonstrated *Sox2ot* expression in NPs, and identified a novel role for *Sox2ot* in repression of NP expansion. Our result is distinct from studies of *Sox2ot* in cancers, where it is found to positively regulate *Sox2* expression^42,43^. Though the cancer studies did not explore the mechanism of *Sox2ot*, one possible explanation for the opposing effects on *Sox2* is that *Sox2* must be reactivated, often by amplification, in cancers, whereas *Sox2* is already highly expressed in NPs in the cortex^44^. Thus, the transcriptional and epigenetic landscape surrounding the *Sox2* locus may look very different and requires different regulation in cancer cells versus NPs.

lncRNAs have been shown to work through a variety of mechanisms, including as scaffolds for ribonucleoprotein complexes, activators of distinct transcriptional programs, and recruiters of proteins to specific loci^45^. Our results support *Sox2ot* action through epigenetic regulation by recruiting the binding partner YY1 to the *Sox2* locus. YY1 is a ubiquitously expressed, multifunctional polycomb group transcription factor, can be activating or repressive depending on the context, and often works by binding CpG islands and recruiting cofactors to perform epigenetic modifications^46^. Studies of YY1 in early nervous system development have demonstrated its importance for neurulation and proper patterning, but its role in cortical development has not been characterized^33,47^. Here, we demonstrate a new prodifferentiative role of YY1 in NPs, as its knockdown results in increased NPs and decreased neurons.

We propose that the function of the *Sox2ot*–YY1 complex is to modulate *Sox2* expression in NPs, helping to balance progenitor pool maintenance with neurogenesis, such that low levels of *Sox2ot* allow high *Sox2* expression, thus promoting proliferation (Supplementary Figure S7A), while higher *Sox2ot* expression represses *Sox2*, resulting in differentiation of neurons (Supplementary Figure S7B). There are several proposed models of YY1-mediated repression, including passively blocking activation by covering DNA recognition sites or interfering with activation factor binding, or actively repressing transcription through co-repressor recruitment^48^. Mechanistically, we propose that low *Sox2ot* expression allows transcription from the *Sox2* locus (Supplementary Figure S7C), while higher expression of *Sox2ot* allows recruitment of YY1, likely with cofactors, to the CpG islands at the *Sox2* locus (Supplementary Figure S7D). Since YY1 binding sites are pervasive throughout mammalian genomes, *Sox2ot* is potentially important for preferentially recruiting YY1 to the *Sox2* locus through *cis*-regulation of *Sox2ot* to its overlapping gene *Sox2*^49,50^. Moreover, the *Sox2ot*–YY1 complex perhaps also binds to promoters of other genes beside of *Sox2*. Identification of other YY1 cofactors in the lncRNA-YY1 system would give further insight into the method of YY1-mediated repression occurring in brain development.

In recent years, lncRNAs as a group have been shown to be expressed in specific and regulated patterns in the brain, important for neural fate specification, and critical for the regulation of neurogenesis^9,51^. Specific lncRNAs have also demonstrated functions in neural development, including *RMST*, which is required for Sox2 binding to neurogenic transcription factors in neural stem cells, thus promoting neurogenesis, and *TUNA*, which is required for pluripotency and neural lineage commitment of mouse ES cells^52,53^. In the embryonic mouse cortex, the lncRNA *Pinky* interacts with RNA-splicing protein PTBP1 to regulate neurogenesis^54^. Finally, *Paupar* induces neural differentiation through both Pax6-dependent and Pax6-independent processes^35^.

In this study, we have characterized the expression of *Sox2ot* in NPs in the developing mouse cortex and established its role in repression of NP expansion. We also demonstrated a novel interaction of *Sox2ot* with the multifunctional transcription factor YY1 and showed that YY1 binds to CpG islands at the *Sox2* locus in neuroectodermal cells. Through this work, we have provided a further example of one lncRNA important in neural development, gained insight into the modes of NP regulation, and expanded the understanding of lncRNA regulatory mechanisms.

## Materials and methods

### In situ hybridization

In situ hybridization was performed according to previously published methods^55^. Briefly, brain sections were hybridized with DIG-labeled RNA probes at 65 °C overnight. After washing with preheated wash solution (1×SSC, 50% formamide, 0.1% Tween-20) and MABT, sections were blocked with blocking buffer (1×MABT, 2% blocking reagent, 20% heat-inactivated sheep serum) and incubated with anti-DIG antibody (1:1,500, Roche) at 4 °C overnight. Brain sections were washed with 1×MABT and staining buffer (0.1 M NaCl, 50 mM MgCl_2_, and 0.1 M Tris–HCl, pH 9.5), and stained with BM Purple (Roche) at room temperature until ideal intensity. The probes were labeled with DIG-ddUTP using the DIG RNA labeling kit (Roche). The images of in situ hybridization were collected using a Leica digital camera under a dissection microscope (Leica, MZ16F).

### In utero electroporation

In utero electroporation was performed as described previously^56^. Briefly, electroporation was conducted at E13.5, and the brain tissues were collected either 24 h later at E14.5 or 96 h later at E17.5. Bromodeoxyuridine (BrdU, 50 µg/g body weight) was administered intraperitoneally as a single pulse 23 h after electroporation, 1 h before sacrifice. Plasmid DNA was prepared using the EndoFree Plasmid Maxi Kit (Qiagen) and diluted to 2 µg/µL. DNA solution was injected into the lateral ventricle of the cerebral cortex and electroporated with five 50-ms pulses at 35 V using an ECM830 electrosquareporator (BTX).

### Tissue preparation and immunohistochemistry

Mouse brains were fixed in 4% paraformaldehyde (PFA) in phosphate-buffered saline (PBS) overnight, incubated in 30% sucrose in PBS, embedded in OCT, and stored at –80 °C until use. Brains were sectioned (14 µm) using a cryostat. For antigen recovery, sections were incubated in heated (95–100 °C) antigen recovery solution (1 mM EDTA and 5 mM Tris, pH 8.0) for 20 min and cooled for 30 min. Before applying antibodies, sections were blocked in normal goat serum (NGS) in PBS with 0.1% Tween-20 for 1 h at room temperature. Sections were incubated with primary antibodies overnight at 4 °C and visualized using goat anti-rabbit IgG-Alexa-Fluor-488 or goat anti-chicken IgG-Alexa-Fluor-488 and goat anti-rabbit IgG Alexa-Fluor-546 or goat anti-mouse IgG-Alexa-Fluor-546 (1:1,000, Molecular Probes) for 1 h at room temperature. For sections stained with Sox2 primary antibody, normal donkey serum was substituted for NGS and donkey anti-rabbit IgG-Alexa-Fluor-488 and donkey anti-goat IgG-Alexa-Fluor-546 were used for visualization (1:1000, Molecular Probes).

Primary antibodies against the following antigens were used: Sox2 (1:200, Santa Cruz), Pax6 (1:500, Covance), Tbr2 (1:500, Abcam), BrdU (1:50, DSHB), Caspase3 (1:1000, R&D Systems), Tbr1 (1:500, Abcam), Satb2 (1:500, Abcam), GFP (1:1000, Rockland, rabbit), GFP (1:1000, Abcam, chicken), and Yy1 (1:200, Santa Cruz).

Images were captured using a Zeiss confocal microscope.

### Quantitation of immunostained tissue

Coronal sections were collected in the medial cortical region. At least four sections from each brain and three electroporated brains from the same litter were chosen for antibody labeling. For neural progenitor markers (Sox2, BrdU, Pax6, and Tbr2), positive cells were quantified in fixed areas of 100 × 100 µm^2^, starting at the ventricular surface. For mature neuronal markers (Tbr1 and Satb2), cells were quantified in columns with a width of 200 µm and height from the lowest point of marker staining to the pial surface.

Cell counting in the mouse brain sections was performed on a fixed width (200-μm bin) of a representative column in the cortical wall. All sections analyzed were selected from a similar medial point on the anterior–posterior axis. Cell counting was performed in minimal three chosen areas in each brain, and at least three brains were analyzed in each group. Cell counting in each chosen area was repeated at least three times and a mean was obtained. All data are presented as mean±SEM. *P* values were calculated using an unpaired Student’s *t* test.

### Embryonic stem cell culture

Stem cell culture and JQ1 treatment were performed, as described previously^29^. Briefly, R1 and LF2 ES cells were maintained for 4 days in DME and 15% FCS. Cells were treated for 24 h with JQ1 (BPS Biosciences) diluted to 2 µM in DMSO.

### RNA immunoprecipitation

RNA immunoprecipitation was performed following the Abcam RIP protocol. Briefly, cells were cross-linked with a final concentration of 0.75% formaldehyde, harvested by trypsinization, and resuspended in PBS. The nuclei were pelleted by centrifugation and resuspended in RIP buffer (150 mM KCl, 25 mM Tris, pH 7.4, 5 mM EDTA, 0.5 mM DTT, 0.5% NP40, and 100 U/mL RNase inhibitor). Chromatin was sheared by sonication, and nuclear membranes were pelleted by centrifugation. For each IP, 4 µg of antibody (YY1, Santa Cruz; CTCF, Santa Cruz; Pax6, Covance) was added, and the samples were incubated overnight at 4 °C with rotation. Protein A/G PLUS-agarose beads (Santa Cruz Biotechnology) were added, and the samples were incubated at 4 °C for 2 h with rotation. The beads were pelleted by centrifugation, resuspended, and washed in RIP buffer. RNA was eluted in RIP elution buffer (50 mM Tris, pH 8, 10 mM EDTA, and 1% SDS) supplemented with proteinase K and RNase inhibitor overnight at 65 °C. Samples were treated with DNase and precipitated overnight.

### Statistics

For immunostaining, at least four sections from each brain and at least three different brains from the same litter were chosen for antibody labeling and quantification. For qRT-PCR, triplicated samples were tested. Statistical comparisons were made by analysis of variance (unpaired Student’s *t* test).

## Electronic supplementary material

Supplementary methods and Figures
