# Supplementary material for: Long noncoding RNA Sox2ot and transcription factor YY1 co-regulate the differentiation of cortical neural progenitors by repressing Sox2
Source: Cell Death Dis. 2018 Jul 23;9(8):799. doi: 10.1038/s41419-018-0840-2 (PMC6056501; doi:10.1038/s41419-018-0840-2)
Supplement: Supplementary file 1 — Supplementary methods and Figures [file 41419_2018_840_MOESM1_ESM.docx]

**Supplementary data:**

**Long noncoding RNA *Sox2ot* and transcription factor YY1 co-regulate differentiation of cortical neural progenitors by repressing *Sox2***

Jennifer L. Knauss^1^, Nan Miao^2^, Seung-Nam Kim^1,6^, Yanzhen Nie^3^, Yuelin Shi^3^, Tao Wu^4,5^, Hugo Borges Pinto^4,5^, Mary E. Donohoe^4,5^ and Tao Sun^1,2,*^

^1^Department of Cell and Developmental Biology, Cornell University Weill Medical College, 1300 York Avenue, Box 60, New York, NY 10065, USA

^2^Center for Precision Medicine, School of Medicine and School of Biomedical Sciences, Huaqiao University, Xiamen, China 361021

^3^School of Life Sciences and Technology, Shanghai Jiao Tong University, Shanghai, China 200240

^4^Burke Medical Research Institute, 785 Mamaroneck Avenue, White Plains, NY 10605, USA

^5^Department of Neuroscience, Department of Cell and Developmental Biology, Cornell University Weill Medical College, 1300 York Avenue, New York, NY 10065, USA

^6^College of Korean Medicine, Dongguk University, Ilsandonggu, Goyangsi 10326, Gyeonggido, Korea

^*^Corresponding author: Dr. Tao Sun, E-mail: taosun@hqu.edu.cn

**Abbreviated title:** *Sox2ot* represses neural progenitor expansion

**Key words:** long noncoding RNA (lncRNA), *Sox2ot*, *Sox2*, YY1, neurogenesis, cerebral cortex

**Supplementary Materials and Methods**

**Animal use**

Transgenic animals were maintained at the facility of Weill Cornell Medical College. Animal use was overseen by the Animal Facility at the Weill Cornell Medical College. The experimental procedure was performed in accordance with relevant guidelines and regulations of the IACUC, and was approved by the IACUC at the Weill Cornell Medical College. The experimental procedure also was in accordance with guidelines of the National Institutes of Health.

**Droplet digital quantitative PCR (ddPCR)**

The ddPCR was conducted using a QX100 Droplet Digital PCR System (Bio-Rad, California, USA). The reaction was performed using 2xQX200 ddPCR EvaGreen Supermix according to the manufacturer's protocol (Bio-Rad, California, USA). A no-template control was used in every ddPCR batch. The sequences of specific primers and probe used to amplify the mouse *Sox2* (Genebank ID: BC057574.1) were as following: forward: 5’- CAACCGATGCACCGCTAC-3’, reverse: 5’- GCGAGTAGGACATGCTGTAG -3’ and for Taqman probe：5’- TCGCAGACCTACATGAACGGCTCGCC -3’ (product size: 103 bp). For the mouse *Sox2ot* (Genebank ID: BC057611.1) were as follows: forward: 5’- AACTGCTACAAGACAACACC -3’, reverse: 5’- GTGTACCAGCTGCAGAGATT -3’ and for Taqman probe：5’- ATGGTCGCCGCGGGTCCAAGCC -3’ (product size: 90 bp). The ddPCR procedure was carried out under the following conditions: initial denaturation at 95°C for 5 min followed by 35 cycles of 15 sec at 95.0°C, 30 sec at 55.3°C, and 5 min at 4°C, and finally, 5 min at 90°C for signal stabilization. The quantification data were analyzed with QuantaSoft software version 1.7.4 (Bio-Rad, California, USA). The results were presented as IS6110 copy number per ul cDNA sample.

**Preparation of *in situ* hybridization probes**

*Sox2ot* and *Sox2* DIG-labeled RNA probes were transcribed from IMAGE clones (Thermo Scientific). For *Sox2ot* antisense probe, the IMAGE clone was linearized with BamHI (NEB) and transcribed using T3 RNA polymerase. For *Sox2ot* sense probe, the IMAGE clone was linearized with EcoRV (NEB) and transcribed using T7 RNA polymerase. For *Sox2* antisense probe, the clone was linearized with NcoI (NEB) and transcribed with T3 RNA polymerase. For *Sox2* sense probe, the clone was linearized with NcoI (NEB) and transcribed with T7 RNA polymerase.

**RNA fluorescence *in situ* hybridization (RNA-FISH)**

*Sox2ot* probes for RNA-FISH assay were synthesized according the following sequence: CUUGGACCCGCGGCGACCAUGCCAGAUCAG. Cultured neural stem cells were fixed with 4% formaldehyde/10% acetic acid and stored overnight in 70% ethanol. The fluorescence-labeled single-strand probes were then hybridized. After labeling, fluorescence signals were detected using a Leica confocal microscope.

**Neural progenitor cultures**

The dorsal cortex of E12.5 mouse embryos was dissected and treated with 0.05% trypsin for 5 min in 37°C to obtain a single cell suspension. Cells were seeded into 24-well plates at the density of 8×10^4^/ml after 24-hour culture. Culture medium contained: DMEM/F12, N2, B27, with fibroblast growth factor 2 (10 ng/ml; Invitrogen).

**Northern blot analysis**

Total RNA was isolated from the dorsal cortices of E13.5 mice using Trizol reagent (Invitrogen) according to manufacturer’s instructions, and all samples were treated with DNase I (NEB) to remove genomic DNA. RNA samples were denatured at 70°C for 10 min and cooled on ice. Millenium RNA Markers (Life Technologies) were denatured according to manufacturer instructions and ethidium bromide was added to a final concentration of 50µg/mL for visualization. Samples were loaded onto a 1% formaldehyde agarose gel and separated at room temperature. After running, the ladder band locations were marked on the gel.

Samples were transferred onto a nitrocellulose membrane using a semi-dry transfer method overnight. After transfer, the ladder band locations were marked on the membrane. After cross-linking for 4 h at 80°C, the membrane was hybridized at 65°C overnight using a denatured RNA probe (same as *in situ* hybridization probes above). After washing, the RNA was detected using the CDP-star chemiluminescent substrate (Roche).

**Cell fractionation**

Cytoplasm and nuclei were prepared from neuroectodermal cells using a method originally described in ref. (Andersen et al. 2002). Briefly, cells were washed with PBS, resuspended in buffer A (10 mm HEPES-KOH (pH 7.9), 1.5 mm MgCl_2_, 10 mm KCl, 0.5 mm DTT), and homogenized. Homogenized nuclei were pelleted, leaving the cytoplasmic fraction as the supernatant. The nuclear pellet was resuspended in 3 ml of 0.25 m sucrose, 10 mm MgCl_2_; layered over 3 ml of 0.35 m sucrose, 0.5 mm MgCl_2_; and centrifuged. The clean, pelleted nuclei were resuspended in 3 ml of 0.35 m sucrose, 0.5 mm MgCl_2_.

**qRT-PCR**

Total RNA was isolated from the dorsal cortices of mice using RNeasy Mini Kit (Qiagen) according to manufacturer’s instructions, and all samples were treated with DNase to remove genomic DNA. For cell culture samples, RNA was isolated using Trizol reagent (Invitrogen), according to manufacturer’s instructions. Reverse transcription was performed using Random Hexamer primers (Roche). The qRT-PCR was performed using Power SYBR Green PCR Master Mix (Life Science) and Mx4000 Multiplex Quantitative PCR System (Stratagene) according to manufacturer’s instructions. Primer sequences are as follows:

*Sox2ot*

F: 5’-TGCTACAAGACAACACCCTGA-3’

R: 5’- GTTGCCTGGCTTCTCTTTTG-3’

*Sox2*

F: 5’- TACAGCATGTCCTACTCGCA-3’

R: 5’- TGGAGTGGGAGGAAGAGGTA-3’

*GAPDH*

F: 5’- ACTCCACTCACGGCAAATTC-3’

R: 5’- CTAAGCAGTTGGTGGTGCAG-3′

*Xist*

F: 5’- CAGAGTAGCGAGGACTTGAAGAG-3’

R: 5’- GCTGGTTCGTCTATCTTGTGGG-3’

*Nestin*

F: 5’- AGGTCTCTAGCAGGACAGGA-3’

R: 5’- AGACTTCCTTCTTCCCAGGC-3’

*Ncam1*

F: 5’- TTGTTCAAGCAGACACACCG-3’

R: 5’-TCAGGTTTCAGGCCCATGAT-3’

*Musashi*

F: 5’-GGTCAGCAGTTACATCAGCG-3’

R: 5’-AGGTTAGTAGGGGAGCCAGA-3’

*Tubb3*

F: 5’-GACGAGACCTACTGCATCGA-3’

R: 5’-GAAGAAGTGGAGACGTGGGA-3’

*Map2*

F: 5’-CTTTCCCCTCTGGCTTCTGA-3’

R: 5’-AGCAAGGCATCTTCTCCACT-3’

*En1*

F: 5’-GACTCACAGCAACCCCTAGT-3’

R: 5’-CTGGGACTCATTCAGGCTGA-3’

*Lmx1a*

F: 5’-AGGACCATAAGCGACCCAAA-3’

R: 5’-AGTCTCCCTCACCTTTCTGC-3’

*Foxa2*

F: 5’-AACAGGTCATGCACTACCCA-3’

R: 5’-GCTCTCCCAAAGTCTCCACT-3’

*Pax6*

F: 5’-AGGAACCAGAGAAGACAGGC-3’

R: 5’-CAGGTTGTTTGCCATGGTGA-3’

*Nurr1*

F: 5’-TTCTGTAGACCCCTAGCCCT-3’

R: 5’-ACTGTGTGCTGTCCGTTTTC-3’

*Chat*

F: 5’-TTCTAGCTGTGAGGAGGTGC-3’

R: 5’-GCTCTTCAGGTACCAGGTGT-3’

*NeuroD1*

F: 5’-AGGAGGAGGATCAAAAGCCC-3’

R: 5’-GGGTCTTGGAGTAGCAAGG-3’

*Oct4*

F: 5’-CCTTGCAGCTCAGCCTTAAG-3’

R: 5’- GCGATGTGAGTGATCTGCTG-3’

*Nanog*

F: 5’-CGCCATCACACTGACATGAG-3’

R: 5’-AGAAGAATCAGGGCTGCCTT-3’

*Eras*

F: 5’-CTACTGGAAGGAAGTGGCCA-3’

R: 5’-TATCTGCTGCAACTGGTCCA-3’

*Fgf4*

F: 5’-CACTCCTTAGGTGCTGGGAA-3’

R: 5’-AATCCCATCAGAACAGGGCA-3’

*Dax1*

F: 5’-ACCGTGCTCTTTAACCCAGA-3’

R: 5’-ACTCTCTCTGCATCATCCGG-3’

*Zfp296*

F: 5’-GTCAACTCCAAACTGCCTCG-3’

R: 5’-TGGATTCTGAGATGGGGTCG-3’

*Ecat1*

F: 5’-CCCAGAAGTGTCCTGAGGTT-3’

R: 5’-TCATTGCCTTACTCAGCCCA-3’

*Esg1*

F: 5’-GATGCTTCAGTCCATGGCTG-3’

R: 5’-ACTCGATACACTGGCCTAGC-3’

*Gdf3*

F: 5’-GCCTTATCAACGGCTTCTGG-3’

R: 5’-TGGTCTGGGAGAAGCTGAAG-3’

*Slc2a3*

F: 5’-GTCTGAGGTTGGAAGGCTCT-3’

R: 5’-AAAGGGGTGAGGTCCATCAG-3’

**Chromatin immunoprecipitation**

Chromatin immunoprecipitation (ChIP) was performed as described in ref. (Carey et al. 2009). Briefly, cells were crosslinked with a final concentration of 0.75% formaldehyde, washed with PBS, and collected in a solution of PBS, 1% BSA, and protease inhibitors. Cells were pelleted, resuspended in ChIP lysis buffer (5mM PIPES, 85mM KCl, 0.5% NP40), and the chromatin was sheared by sonication. Chromatin was diluted in ChIP dilution buffer (16.7mM Tris pH 8, 167mM NaCl, 1.2mM EDTA, 1.1% Triton X-100) and precleared with Protein A/G PLUS-agarose beads (Santa Cruz Biotechnology) for 1 hour at 4°C. Chromatin was incubated with 4µg antibody (YY1) overnight at 4°C and immunoprecipitated with Protein A/G beads for 2 hours at 4°C. Samples were washed with ChIP dilution buffer and eluted with ChIP elution buffer (50mM Tris pH8, 10mM EDTA, 1% SDS) supplemented with Proteinase K by incubation for 2 hours at 55°C. The crosslinks were reversed by incubating overnight at 65°C. DNA was purified by phenol:chloroform extraction and quantified by PCR.

For ChIP from electroporated cortical tissues, the electroporated portion of the cortex was dissected and chopped into small pieces. Crosslinking was performed as above, followed by additional tissue breakdown via vortexing and passage through a syringe needle. After this additional tissue breakdown, the ChIP protocol was performed as above.

Primer sequences are as follows:

*Sox2* CpG1

F: 5’-GATGACGTGCCCTCTCATCT-3’

R: 5’-CGAGGCTAGTCTCAGGTTGG-3’

*Sox2* CpG2

F: 5’-CAGAAACAATGGCACACCAC-3’

R: 5’-CAAGACGACAGCTCCTTTCC-3’

*Sox2* CpG3

F: 5’-ATCTGGAGCGTGCTTGATCT-3’

R: 5’-GGGAGACGCAAATATGGAAA-3’

*Sox2* Upstream region 6

F: 5’-GGCAGAGTTGGGGTAGATGA-3’

R: 5’-CCCCGTCTAAGTTTCCTTCC-3’

*Sox2* Upstream region 5

F: 5’-GGTGCAAACACACATTGGAG-3’

R: 5’-ACGAGTTGATGTGGACAACG-3’

*Sox2* Upstream region 4

F: 5’-CTCAAATGCAGATGCAGGAG-3’

R: 5’-GCCAACTGACAATGTTGTGG-3’

*Sox2* Upstream region 3

F: 5’-GAGGAAACCTCACAGCGAAG-3’

R: 5’-AGATGACGACAGGGAAATGC-3’

*Sox2* Upstream region 2

F: 5’-ACTTCCAAGCGTTGATTTGG-3’

R: 5’-TACCACTGCTGTTGCTGTCC-3’

*Sox2* Upstream region 1

F: 5’-AGCCCCAGCTAAGAAGAAGG-3’

R: 5’-GGCAATGTCTGAGAACGTCA-3’

**Constructs**

For *Sox2ot* overexpression, cDNA from a *Sox2ot* IMAGE clone (Thermo Scientific) was inserted into pCAGIG. To make constructs for *Sox2ot* and *YY1* shRNAs, hairpin-forming oligonucleotides were designed and inserted into the pSilencer-IRES-eGFP vector. The sequences of the shRNAs are as follows:

shSox2ot A: 5’-TCCTCAAGATCCGCTTAAG-3’

shSox2ot B: 5’-CTGGAAGAGCTTAGCAACA-3’

shYY1 A:

5’-CCTCTCCTTTGTATATTATTAAGTTCTCTAATAATATACAAAGGAGAGGCTTTTTT-3’

shYY1 B:

5’-ACAGAAAGGGCAACAATAATTCAAGAGATTATTGTTGCCCTTTCTGTTTTTTT-3’

shCtrl:

5’-ATGCACGTGCACATATCCCTTCAAGAGAGGGATATGTGCACGTGCATTTTTT-3’

**References**

Andersen JS, Lyon CE, Fox AH, Leung AKL, Lam YW, Steen H, Mann M, Lamond AI. 2002. Directed proteomic analysis of the human nucleolus. *Curr Biol* **12**: 1–11. http://www.ncbi.nlm.nih.gov/pubmed/11790298.

Carey MF, Peterson CL, Smale ST. 2009. Chromatin Immunoprecipitation (ChIP). *Cold Spring Harb Protoc* **4**: 1–14.

**Figure legends:**

**Supplementary Figure S1.** Characterization of *Sox2* and *Sox2ot* expression.

(**A**) Low power (top panels) and high power (bottom panels) images of *in situ* hybridization in wild type mice at P0. The ventricular zone is labeled (arrowheads).

(**B**) Low power (top panels) and high power (bottom panels) images of *in situ* hybridization with sense probes in wild type mice at E13.5.

(**C** and **D**) Copy numbers of *Sox2ot* and *Sox2* mRNA per µl from the dorsal cortex at various developmental stages, detected by droplet digital quantitative PCR (ddPCR). n=3 repeats.

**Supplementary Figure S2.** Altering *Sox2ot* causes changes in numbers of neural progenitors.

(**A-D**) Electroporation of ectopic *Sox2ot (OE)* at E13.5 for analysis at E14.5 significantly decreased the number of Pax6^+^, or Tbr2^+^ cells co-labeled with GFP in the cortex, compared to controls (*Ctrl*).

(**E** and **F**) Electroporation of ectopic *Sox2ot* at E13.5 for analysis at E14.5 did not significantly change the number of Casp3^+^ cells co-labeled with GFP in the cortex.

Yellow and white cells indicate co-labeled cells. Data are presented as mean ± SD; n ≥ 5 sections from at least 4 different brains; *p* values in relation to the empty vector control (****p* < 0.001, n.s., not significant). Scale bar: 50µm.

**Supplementary Figure S3.** *Sox2ot* manipulation *in vivo* affects neural markers at the RNA level.

**(A)** Relative expression of *Sox2ot* and *Sox2* upon either control plasmid (*Ctrl*) or *Sox2ot OE* electroporation detected by real time RT-PCR.

**(B)** Relative expression of *Pax6*, *Tbr2*, and *Tbr1* upon either *Ctrl* or *Sox2ot OE* electroporation detected by real time RT-PCR.

(**C**) Relative expression of *YY1* upon control plasmid, *Sox2ot OE*, *shSox2ot*, or *shYY1* electroporation detected by real time RT-PCR.

Data are presented as mean ± SD; n = 3 for all real time RT-PCR based on at least 4 different electroporated brains; *p* values in relation to control (****p* < 0.001).

**Supplementary Figure S4.** Characterization of knockdown of *Sox2ot*.

(**A**) Diagram of *Sox2ot* locus demonstrating shRNA targeting locations.

(**B**) Relative expression of *Sox2ot* in Neuro2a cells after transfection of overexpression or shRNA constructs detected by real time RT-PCR.

(**C** and **D**) Relative expression of *Sox2ot*, *Sox2*, *Pax6*, *Tbr2*, and *Tbr1* upon control plasmid, *shSox2ot*, or *shYY1* electroporation detected by real time RT-PCR.

Data are presented as mean ± SD; n = 3 for all real time RT-PCR; *p* values in relation to control (**p* < 0.05, ***p* < 0.01, ****p* < 0.001, n.s., not significant).

**Supplementary Figure S5.** Altering *Sox2ot* causes changes in numbers of neural progenitors.

(**A-D**) Electroporation of shRNAs against *Sox2ot* (*shSox2ot A* and *shSox2ot B*) at E13.5 for analysis at E14.5 significantly increased the number of Pax6^+^, or Tbr2^+^ cells co-labeled with GFP in the cortex.

Yellow and white cells indicate co-labeled cells. Data are presented as mean ± SD; n ≥ 5 sections from at least 4 different brains; *p* values in relation to the empty vector control (***p* < 0.01, ****p* < 0.001). Scale bar: 50µm.

**Supplementary Figure S6.** YY1 binds *Sox2* CpG islands in neuroectodermal cells.

Chromatin immunoprecipitation (ChIP) with α-YY1 or control antibody (IgG) from dissected electroporated region of mouse cortices that were electroporated with the control (Ctrl) vector. Relative enrichment of Sox2 locus locations detected by real time RT-PCT.

Data are presented as mean ± SD; n = 3 different brain samples for all real time RT-PCR; *p* values in relation to the control.

**Supplementary Figure S7.** Proposed models for *Sox2ot* function and mechanism.

(**A** and **B**) Proposed biological function of *Sox2ot* in neural progenitors. When *Sox2ot* expression is low (**A**), Sox2 promotes an expansion of the neural progenitor pool by the proliferation of radial glia cells and intermediate progenitors in the ventricular zone (VZ) and subventricular zone (SVZ), respectively. When *Sox2ot* expression is elevated (**B**), it represses *Sox2*, thereby repressing progenitor expansion and promoting neuronal differentiation in the cortical plate (CP).

(**C** and **D**) Proposed mechanism of *Sox2ot* in neural progenitors. When *Sox2ot* expression is low (**C**), *Sox2* is robustly expressed. Upon an increase in *Sox2ot* expression (**D**), YY1 is recruited to the CpG islands at the *Sox2* locus; YY1 and its cofactors perform epigenetic regulations to these CpG islands, subsequently decreasing *Sox2* expression.

**Figure S1**

**
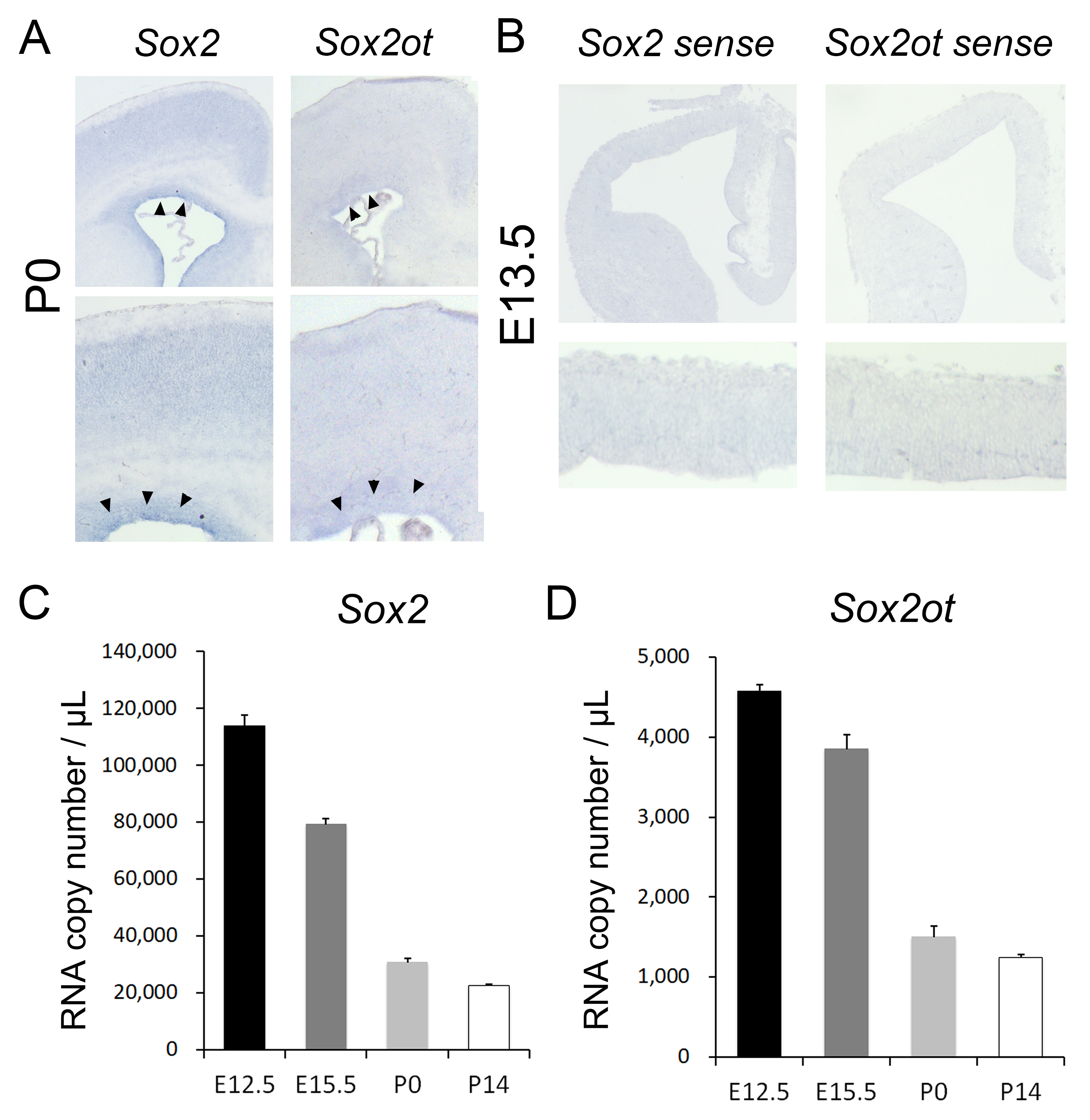
**

**Figure S2**

**
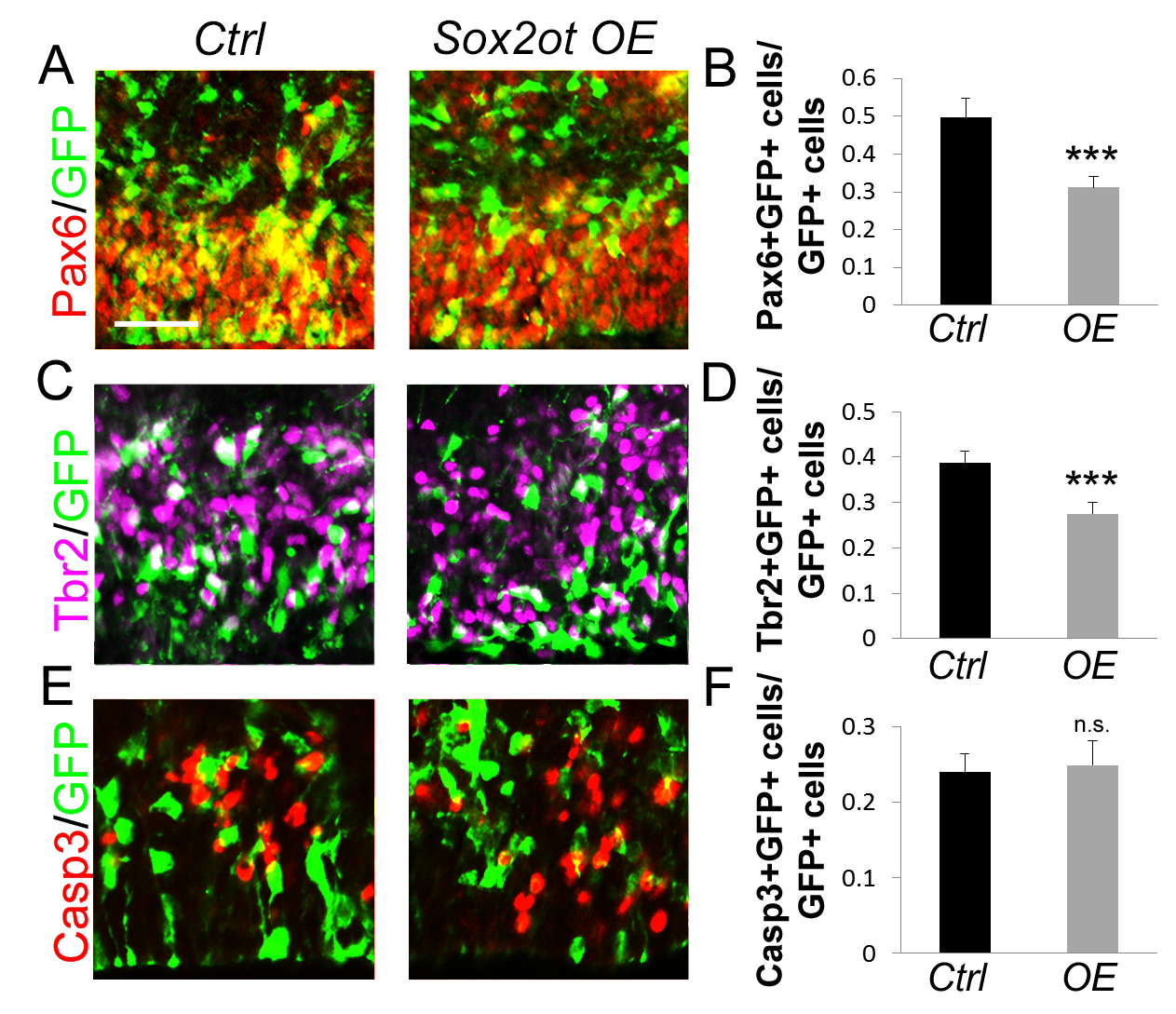
**

**Figure S3**

**
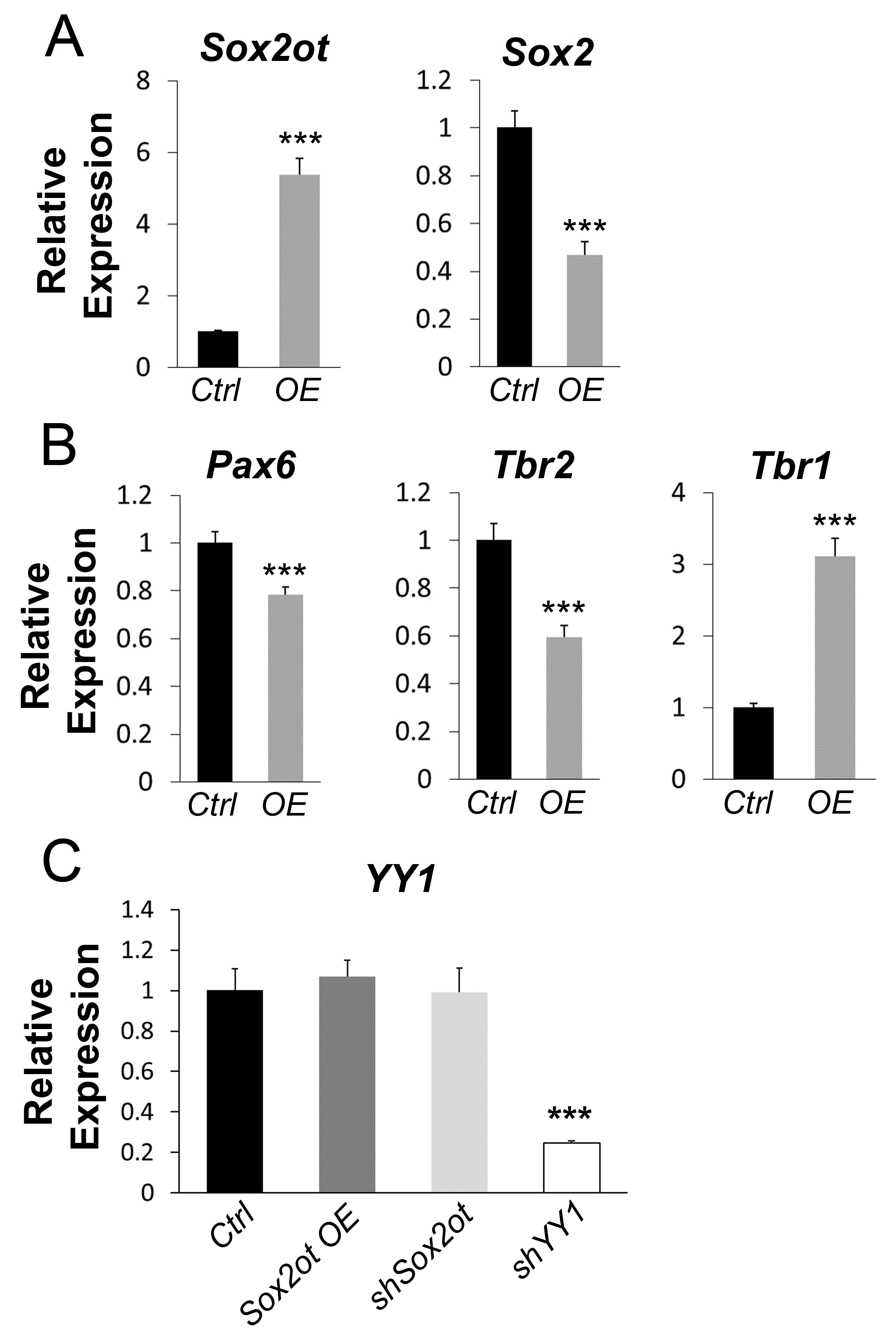
**

**Figure S4**

**
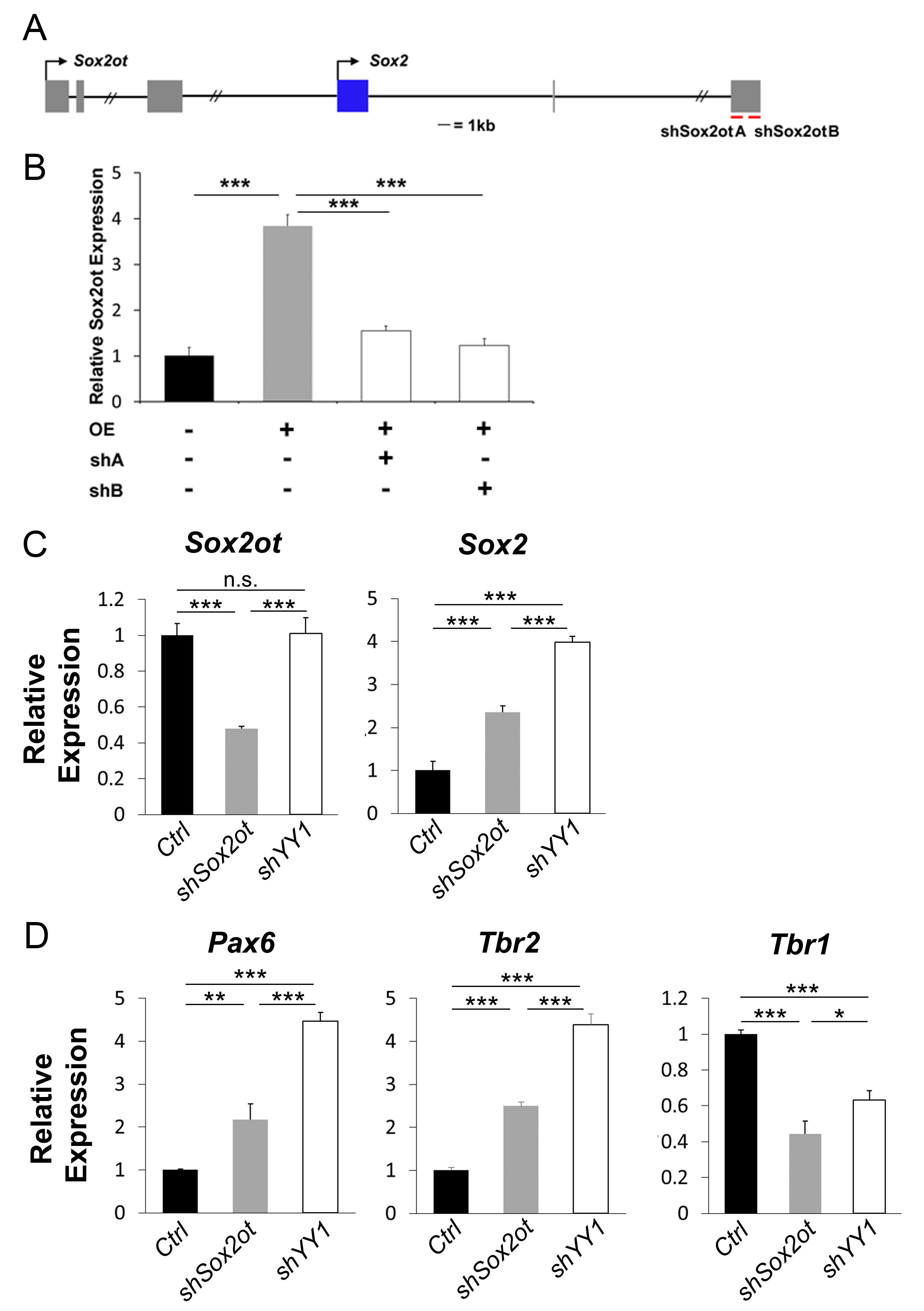
**

**Figure S5**

**
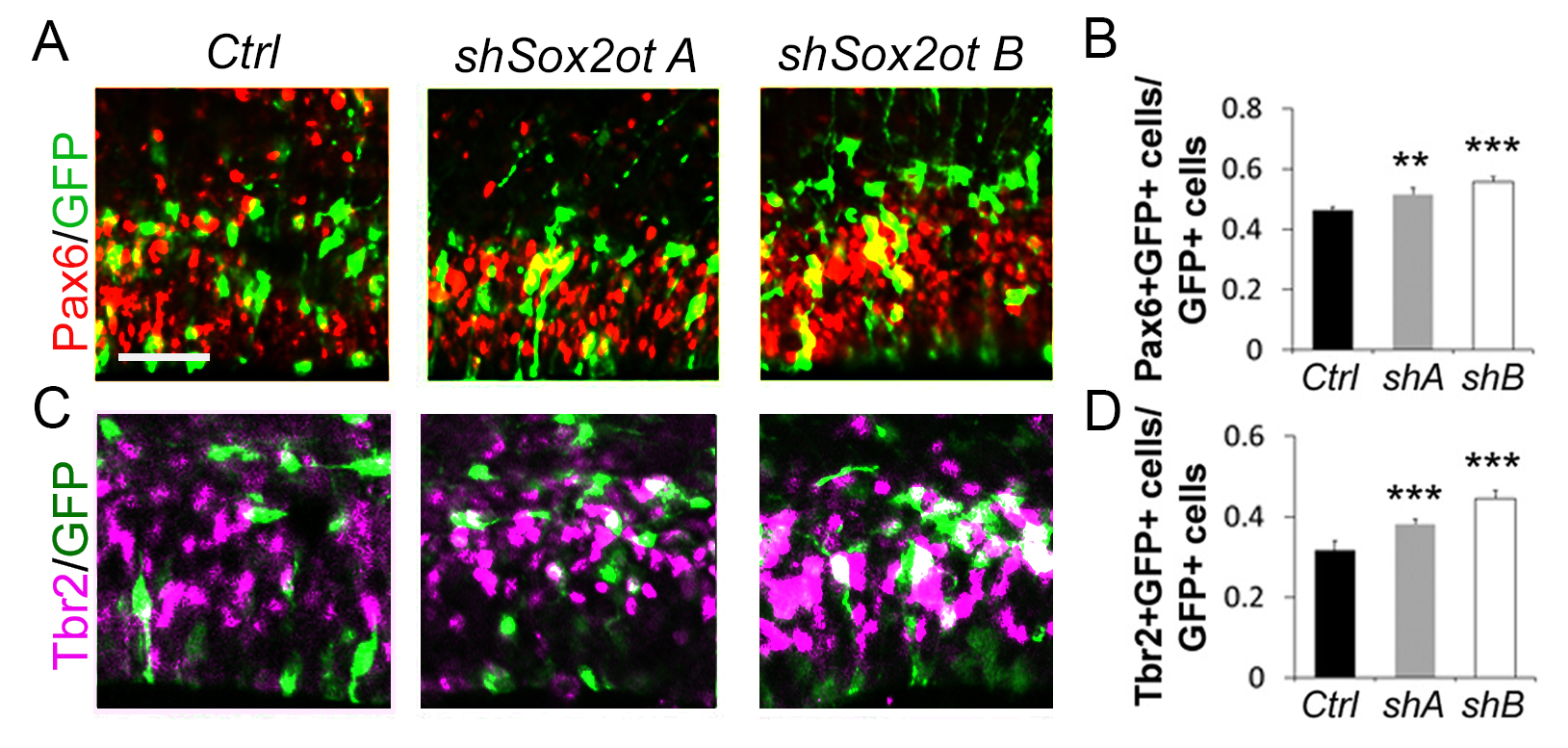
**

**Figure S6**

**
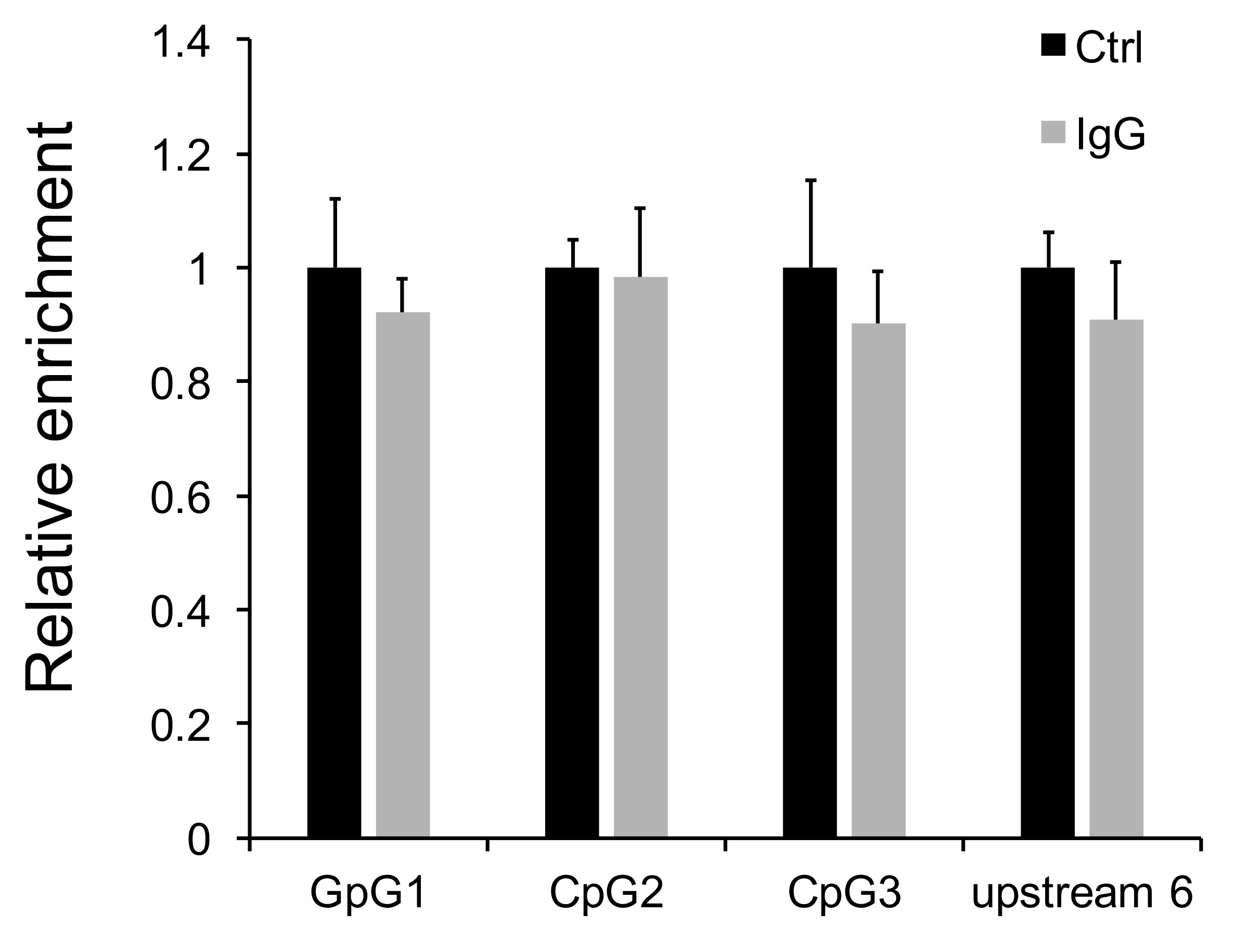
**

**Figure S7**

**
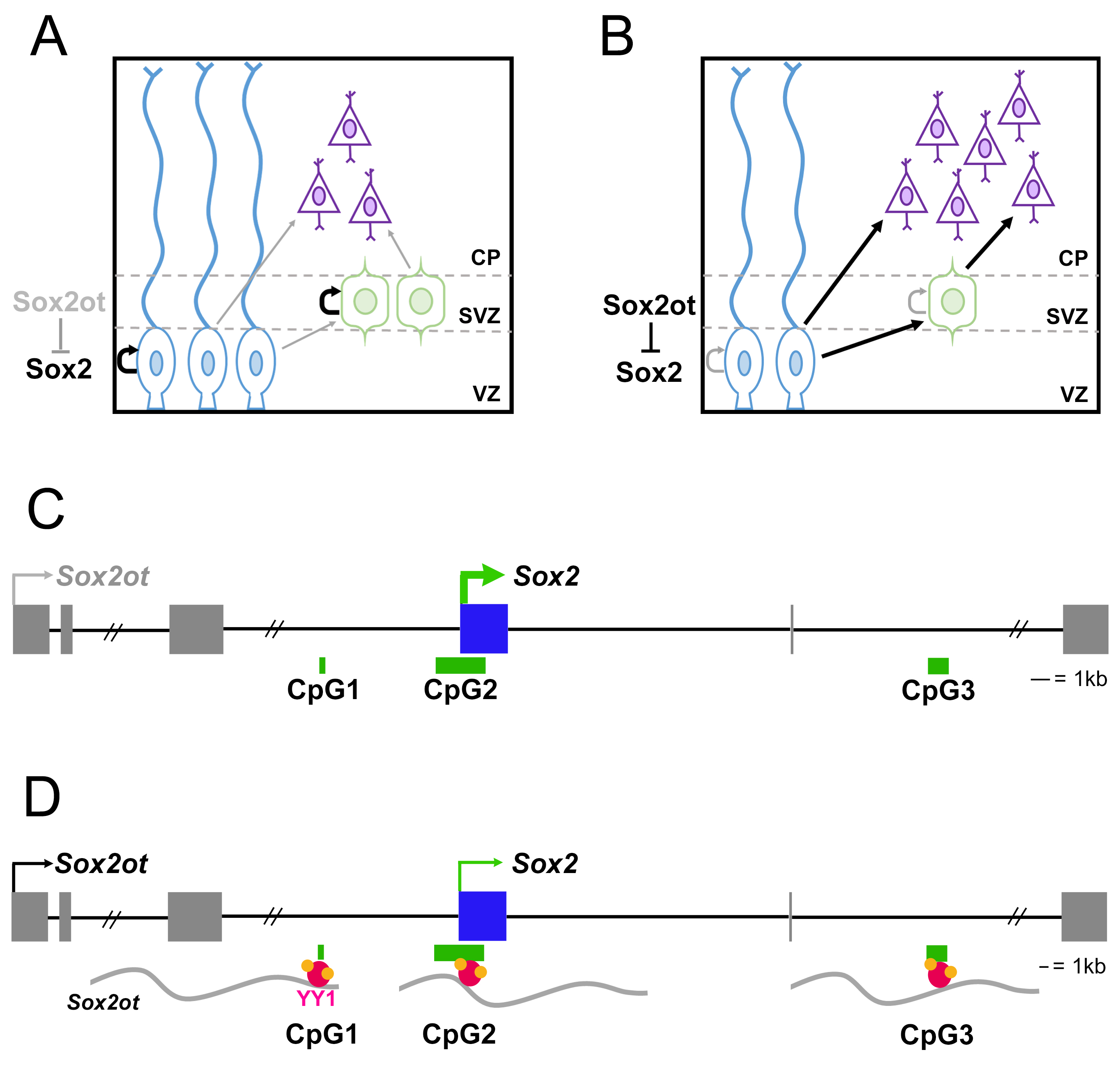
**
